# The *Arabidopsis* IDD14, IDD15, and IDD16 Cooperatively Regulate Lateral Organ Morphogenesis and Gravitropism by Promoting Auxin Biosynthesis and Transport

**DOI:** 10.1371/journal.pgen.1003759

**Published:** 2013-09-05

**Authors:** Dayong Cui, Jingbo Zhao, Yanjun Jing, Mingzhu Fan, Jing Liu, Zhicai Wang, Wei Xin, Yuxin Hu

**Affiliations:** 1 Key Laboratory of Plant Molecular Physiology, Institute of Botany, Chinese Academy of Sciences, Beijing, China; 2 University of Chinese Academy of Sciences, Beijing, China; 3 National Center for Plant Gene Research, Beijing, China; National University of Singapore and Temasek Life Sciences Laboratory, Singapore

## Abstract

The plant hormone auxin plays a critical role in regulating various aspects of plant growth and development, and the spatial accumulation of auxin within organs, which is primarily attributable to local auxin biosynthesis and polar transport, is largely responsible for lateral organ morphogenesis and the establishment of plant architecture. Here, we show that three *Arabidopsis* INDETERMINATE DOMAIN (IDD) transcription factors, IDD14, IDD15, and IDD16, cooperatively regulate auxin biosynthesis and transport and thus aerial organ morphogenesis and gravitropic responses. Gain-of-function of each *IDD* gene in *Arabidopsis* results in small and transversally down-curled leaves, whereas loss-of-function of these *IDD* genes causes pleiotropic phenotypes in aerial organs and defects in gravitropic responses, including altered leaf shape, flower development, fertility, and plant architecture. Further analyses indicate that these *IDD* genes regulate spatial auxin accumulation by directly targeting *YUCCA5* (*YUC5*), *TRYPTOPHAN AMINOTRANSFERASE of ARABIDOPSIS1* (*TAA1*), and *PIN-FORMED1* (*PIN1*) to promote auxin biosynthesis and transport. Moreover, mutation or ectopic expression of *YUC* suppresses the organ morphogenic phenotype and partially restores the gravitropic responses in gain- or loss-of-function *idd* mutants, respectively. Taken together, our results reveal that a subfamily of IDD transcription factors plays a critical role in the regulation of spatial auxin accumulation, thereby controlling organ morphogenesis and gravitropic responses in plants.

## Author Summary

Auxin is a key plant hormone and the spatial accumulation of auxin is essential for lateral organ morphogenesis and gravitropic responses in higher plants. However, the various mechanisms through which spatial auxin accumulation is regulated remain to be fully elucidated. Here, we identify a gain-of-function mutant of *Arabidopsis IDD14* that exhibits small and transversally down-curled leaves. Further characterization of both gain- and loss-of-function mutants in *IDD14* and its close homologs, *IDD15* and *IDD16*, reveals that these three IDD transcription factors function redundantly and cooperatively in the regulation of multiple aspects of lateral organ morphogenesis and gravitropic responses. We further demonstrate that these IDD transcription factors influence the spatial accumulation of auxin by directly targeting auxin biosynthetic and transport genes to activate their expression. These findings identify a subfamily of IDD transcription factors that coordinates spatial auxin gradients and thus directs lateral organ morphogenesis and gravitropic responses in plants.

## Introduction

Auxin is a key plant hormone that plays critical roles in the regulation of plant growth and development. A combination of physiological, genetic, biochemical, and molecular studies has greatly enriched our understanding of auxin biosynthesis, transport, and signal transduction [1]–[3]. Increasing evidence indicates that auxin is essential for nearly all developmental processes, including gametogenesis, embryogenesis, lateral organ formation and patterning, branching, and tropic responses [4], [5]. It is generally believed that most auxin-mediated developmental events are highly dependent on the differential accumulation of auxin within plant organs (auxin gradients), which are mainly attributable to both local auxin biosynthesis and the intercellular polar transport of auxin [1], [5].

Direct evidence that local auxin biosynthesis is involved in the regulation of plant organogenesis comes from studies of several genes in the auxin biosynthetic pathway of *Arabidopsis*, including *YUCCA* (*YUC*) and *TRYPTOPHAN AMINOTRANSFERASE of ARABIDOPSIS* (*TAA*). *YUC*s encode the flavin monooxygenases that catalyze a key step in converting tryptophan into IAA, a main auxin in plants [6]. Overexpression of *YUC* genes in *Arabidopsis* substantially elevates the endogenous IAA level and causes distinct phenotypes such as epinastic cotyledons, elongated hypocotyls, and narrow and curly leaves [6]–[8]. Although a *yuc* single mutant in *Arabidopsis* does not show an obvious phenotype, the mutation of multiple *YUC* genes leads to a diversity of auxin-related phenotypes, including reduced apical dominance, crinkled leaves, simple venation, and abnormal flower development, demonstrating that YUC-modulated local auxin biosynthesis is critical for plant morphogenesis and architecture formation [9], [10]. Consistently, such developmental defects in *yuc* mutants can be rescued by local expression of *iaaM*, a bacterial auxin biosynthetic gene, but not by the application of exogenous auxin [9]. The expressions of *YUC* genes are overlapping and spatiotemporally regulated in various organs [9], [10], suggesting that *YUC*s function redundantly and cooperatively in different organs. Further studies demonstrate that *TAA1* and its homologs function in auxin biosynthesis in response to environmental and developmental signals in *Arabidopsis*. The *taa1* plant has a decreased level of endogenous IAA and displays defects in shade avoidance and root-specific ethylene sensitivity, and the simultaneous mutation of *TAA1* and its close homologs (*TAR1* and *TAR2*) results in phenotypes that are obviously auxin-related, such as reduced gravitropic response of roots, shortened hypocotyls, and *monopteros-*like seedlings with a single cotyledon [11]–[13]. Recently, YUCs and TAAs have been shown to function in a key two-step auxin biosynthetic pathway [14]–[16]. Therefore, the regulation of YUCs and TAAs may represent an important mechanism to alter auxin gradients and thus modify plant morphogenesis and responses to environmental cues. Indeed, several transcription factors have been shown to participate in the regulation of *YUC* expression. STYLISH1 (STY1), one of the SHORT INTERNODES (SHI) transcription factors and NGATHA3 (NGA3), a member of the B3 transcription factor family, are both proposed to act cooperatively to direct style development, partially through the activation of *YUC2-* and *YUC4*-mediated auxin biosynthesis in the apex of the gynoecium [17], [18]. LEAFY COTYLEDON2 (LEC2), a B3 domain transcription factor acting as a central regulator of embryogenesis, has been found to be involved in modulating *de novo* auxin biosynthesis via the activation of *YUC2* and *YUC4* [19]. Recent studies have also revealed that PIF4-activated *YUC8* expression is required for hypocotyl growth under high temperature conditions [20], and a putative transcription factor SPOROCYTELESS/NOZZLE (SPL/NZZ) has been found to be involved in the regulation of lateral organ morphogenesis by repressing transcription of *YUC2* and *YUC6* [21].

In addition to local auxin biosynthesis, polar auxin transport also contributes greatly to auxin redistribution. The polar auxin transport is mediated by AUX1/LIKE AUX1 (AUX1/LAX), PIN-FORMED (PIN), and ATP-binding cassette class B (ABCB) transporters [2], [22]–[24]. Mutation in *AUX1/LAX*, which encode auxin influx carriers, influences lateral root formation, root hair development, phyllotaxy, and tropic responses [22], [24]–[28]. Similarly, single or multiple mutations in auxin efflux carrier genes, *PIN*s and *ABCB*s, lead to severe defects in multiple developmental processes including embryogenesis, lateral organ formation and patterning, vascular development, and tropic responses [29]–[33], demonstrating that polar auxin transport is also essential for plant morphogenesis and tropic responses.

The INDETERMINATE DOMAIN (IDD) transcription factors belong to a plant-specific transcription factor family that contains a conserved ID domain with four zinc finger motifs [34], [35]. The founding member of this family, maize *INDETERMINATE1* (*ID1*), is a key regulator of flowering transition [36]. The *Arabidopsis* genome contains 16 IDD members, and the characterization of several IDD members in *Arabidopsis* has indicated that *IDD* genes are involved in regulation of multiple developmental processes. For example, *IDD8/NUTCRACKER* (*NUC*) is involved in the regulation of flowering time through modulation of sugar metabolism [37], and *IDD14* regulates starch metabolism in response to cold stimulus [38]. *IDD15/SHOOT GRAVITROPISM 5* (*SGR5*) is involved in the gravitropic response of inflorescence stems, possibly through alteration of gravity sensing [39]–[41]. *IDD8/NUC*, *IDD3/MAGPIE* (*MAG*), and *IDD10/JACKDAW* (*JKD*) regulate root development and patterning [42], [43], while *IDD1/ENHYDROUS* (*ENY*) participates in seed maturation and germination [44]. Recent studies have demonstrated that a rice ID1 homolog is required for flowering induction [45]–[47], and that the rice *Loose Plant Architecture1* (*LPA1*), an ortholog of *Arabidopsis IDD15*, influences shoot gravitropism and architecture [48].

Here, we identified a gain-of-function mutant of *Arabidopsis IDD14*, *idd14-1D*, which exhibited small and transversally down-curled leaves. Further characterization of loss- and gain-of-function mutants of *IDD14* and its close homologs, *IDD15* and *IDD16*, revealed that these three *IDD* genes redundantly but differentially regulate multiple aspects of organ development and gravitropic responses. Moreover, we provide evidence that these IDD transcription factors can directly target auxin biosynthetic and transport genes and alter auxin accumulation in multiple organs. These results reveal that IDD-mediated auxin biosynthesis and transport are critical for lateral organ morphogenesis and gravitropic responses in plants.

## Results

### *cuf1-D*/*idd14-1D* Displays Pleiotropic Leaf Phenotypes

**Figure 1:**
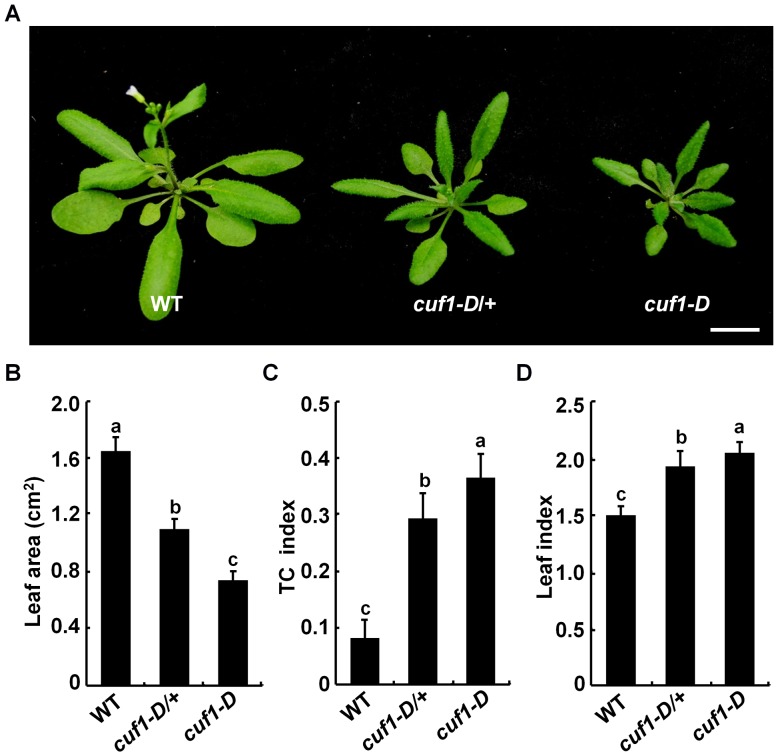
Phenotype of *cuf1-D* plants. (A) Morphology of 4-week-old wild-type (WT), *cuf1-D/+*, and *cuf1-D* plants. The scale bar represents 1 cm. (B) Blade areas of WT, *cuf1-D/+*, and *cuf1-D* leaves. (C) Transverse curvature (TC) index of WT, *cuf1-D/+*, and *cuf1-D* leaves. (D) Leaf index (ratio of length to width) in WT, *cuf1-D/+*, and *cuf1-D* plants. At least 10 sixth leaves from each genotype were used for determination of the leaf area, TC index, and leaf index as described in the methods section, respectively. Data are shown as mean values ± one SD, and the letters (a to c) indicate the significance (P<0.05) among the genotypes according to one-way ANOVA test (SPSS 13.0, Chicago, IL, USA).

To gain insight into how lateral organ morphogenesis is controlled in plants, we screened for mutants with altered aerial organ morphology in a transgenic *Arabidopsis* population harboring a T-DNA activation-tagging plasmid (pSKI015) [49]. A semi-dominant mutant was identified by its smaller and dramatically down-curling leaf phenotype, and thus designated as *curlyfolia1-D* (*cuf1-D*) and subsequently as *idd14-1D* (see below) (Figure 1A). To examine the genetic nature of *cuf1-D*, we backcrossed *cuf1-D* with wild type (WT) plants. All F1 plants exhibited an intermediate phenotype as *cuf1-D/+*, and F2 plants displayed a phenotypic segregation of WT∶*cuf1-D*/+∶*cuf1-D* as 1∶2∶1 (82∶173∶87, P = 0.89, *X^2^-test*), in which all WT plants were BASTA sensitive. This observation suggests that *cuf1-D* results from a semi-dominant mutation of a single gene that is likely to co-segregate with a T-DNA insertion event. The most striking phenotype in *cuf1-D* was the leaf size and shape. Detailed quantification showed that the average areas of fully-expanded leaf blades in heterozygous and homozygous *cuf1-D* were only about 70% and 47% of that in WT, respectively (Figure 1B). The leaves of *cuf1-D* were also dramatically curled downward in a transverse direction. The leaf transverse curvature (TC) index in WT was about 0.08, whereas the leaf TC index in heterozygous and homozygous *cuf1-D* reached about 0.29 and 0.36, respectively (Figure 1C). Moreover, the lamina of a mature WT rosette leaf displayed an elliptical shape with a leaf length/width index at about 1.5, while the heterozygous and homozygous *cuf1-D* leaves were comparatively narrow and their leaf indices were about 1.9 (Figure 1D). In addition to the morphological changes in leaves, *cuf1-D* was also late flowering and dwarfed (Figure 1A, Table S1). These observations demonstrate that the dominant mutation in *cuf1-D* has pleiotropic effects on lateral organ development and plant architecture.

### *CUF1* Encodes the INDETERMINATE DOMAIN 14 Transcription Factor

**Figure 2:**
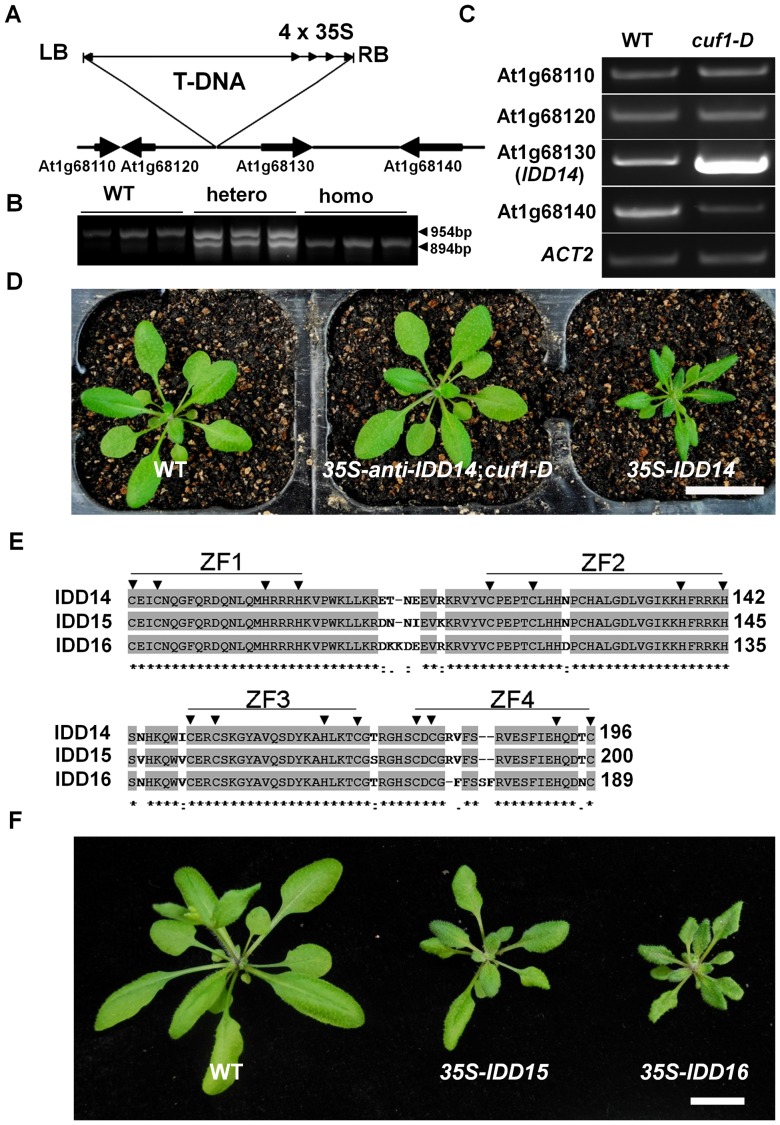
Molecular characterization of *CUF1*/*IDD14*. (A) Scheme of the genomic region flanking T-DNA insertion in *cuf1-D*. Genes are shown as thick arrows and intergenic regions are shown as lines. The orientation of T-DNA left border (LB), right border (RB), and the four CaMV 35S enhancers (4×35S) are indicated. (B) Linkage analysis of the T-DNA and *cuf1-D* phenotype. The 954-bp genomic fragments in WT and *cuf1-D/+* plants were amplified with primers in the genomic region flanking the T-DNA insertion site, and the 894-bp fragments in *cuf1-D/+* and *cuf1-D* were amplified with an LB primer and a downstream genomic primer. (C) Expression analysis of the genes flanking the T-DNA in WT and *cuf1-D* plants. *ACTIN2* (*ACT2*) was used as an internal control. Note that transcripts of At1g68130 (*IDD14*) are highly elevated in *cuf1-D*. (D) Morphology of 25-days-old WT, *cuf1-D* carrying *p35S::anti-IDD14*, and *p35S::IDD14* plants. The scale bar represents 2 cm. (E) Alignment of the ID domains in IDD14, IDD15, and IDD16. ZF1-ZF4 represents the four C2H2-type zinc finger motifs. The arrowheads indicate the conserved cysteine and histidine residues. (F) Morphology of 4-week-old transgenic plants overexpressing *IDD15* or *IDD16*. The scale bar represents 1 cm.

Since *cuf1-D* is a single gene mutation that is likely to co-segregate with a T-DNA insertion event, we amplified the genomic DNA adjacent to the left border of the T-DNA by thermal asymmetric interlaced PCR (TAIL-PCR). The sequencing analysis indicated that a T-DNA was inserted in the intergenic region between At1g68120 and At1g68130 (Figure 2A). Genotyping analysis showed that the T-DNA insertion co-segregated with the leaf phenotype in heterozygous and homozygous *cuf1-D* mutants (Figure 2B), suggesting that *cuf1-D* was caused by the T-DNA insertion event. Because *cuf1-D* was a dominant mutant, we monitored the transcripts of genes flanking the T-DNA insertion using semi-quantitative reverse transcription PCR (RT-PCR) analysis. Compared to those in the WT, the transcripts of At1g68130 (*IDD14*) were dramatically elevated, while At1g68140 mRNA levels were slightly decreased in *cuf1-D* (Figure 2C). To determine whether the elevated levels of *IDD14* transcripts are responsible for the *cuf1-D* phenotype, we introduced a *p35S::anti-IDD14* construct into *cuf1-D* and a *p35S::IDD14* construct into WT plants, respectively. Transgenic plants overexpressing *IDD14* fully recapitulated the phenotype of *cuf1-D*, while the expression level of At1g68140 in these plants was comparable with that in WT plants (Figure 2D, S1B). Moreover, introduction of *p35S::anti-IDD14* into *cuf1-D* restored the *cuf1-D* to WT morphology (Figure 2D). These results demonstrate that *cuf1-D* results from the ectopic expression of *IDD14*, and accordingly, the *cuf1-D* was thus re-designated as *idd14-1D*.

In *Arabidopsis*, the IDD14 transcription factor is phylogenetically sub-grouped with two close homologs: IDD15 (SGR5) and IDD16, to form a small subfamily that is distinct from the other IDD family members (Figure S1A) [34], [39]. IDD14, IDD15 and IDD16 share 52%–62% amino acid identity, and their ID domains are highly conserved with 89%–95% amino acid identity (Figure 2E). To investigate the possible redundancy of *IDD15* and *IDD16* with *IDD14*, we generated transgenic *Arabidopsis* plants overexpressing *IDD15* or *IDD16*, respectively. The ectopic expression of either *IDD15* or *IDD16* resulted in a similar leaf phenotype as observed in *idd14-1D* (Figure 2F, S1B), suggesting that the three IDD members may have redundant function during plant development.

### The Differential and Overlapping Expression of *IDD14*, *IDD15*, and *IDD16* in Multiple Organs

**Figure 3:**
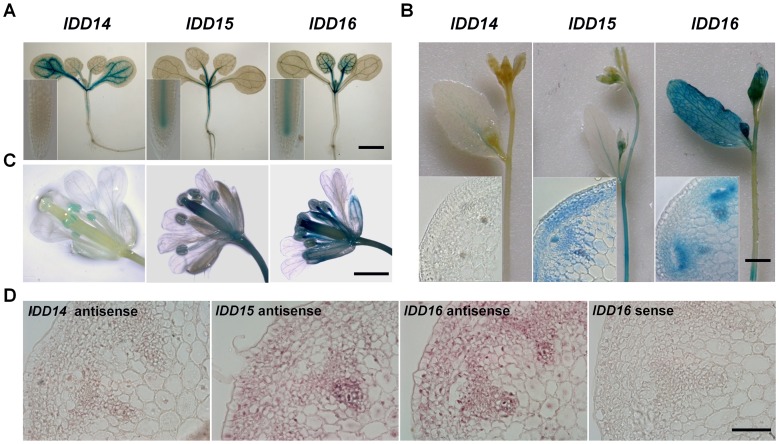
Differential and overlapping expression of *IDD14*, *IDD15*, and *IDD16*. (A) Expression of *IDD14*, *IDD15*, and *IDD16* in seedlings assayed by GUS staining. The 12-day-old transgenic seedlings carrying *pIDD14::GUS*, *pIDD15::GUS*, or *pIDD16::GUS* constructs were subjected to GUS staining assays. Insets show the primary roots. The scale bar represents 1 mm. (B) GUS staining of the florescence stems of *pIDD14::GUS*, *pIDD15::GUS*, and *pIDD16::GUS* transgenic plants. Insets show the transverse sections of inflorescence stems. The scale bar represents 2 mm. (C) GUS staining of the floral organs of *pIDD14::GUS*, *pIDD15::GUS*, and *pIDD16::GUS* transgenic plants. The scale bar represents 1 mm. (D) Expression of *IDD14*, *IDD15*, and *IDD16* in inflorescence stems assayed by RNA *in situ* hybridization. A section hybridized with an *IDD16* sense probe is shown as a control. The scale bar represents 50 µm.

To explore the functions of this *IDD* subfamily, we examined the tissue-specific expression patterns of these genes in multiple organs of transgenic plants harboring a *pIDD*::*GUS* (β-Glucuronidase) construct. As shown in Figure 3A–3C, *IDD14* was mainly expressed in cotyledons and the vasculature of rosette leaves, and a weak level of expression was observed in hypocotyls and floral organs. However, the GUS signal was undetectable in roots and inflorescence stems. *IDD15* was highly expressed in petioles, hypocotyls, roots, floral organs, and especially in inflorescence stems. In inflorescence stems, GUS staining was mainly present in the cortex, endodermis and vasculature tissues (Figure 3A–3C). *IDD16* was highly expressed in leaves, hypocotyls, roots, vasculature of cotyledons, floral organs, and in the endodermis and vasculature of inflorescence stems (Figure 3A–3C). RNA *in situ* hybridization assayed in the inflorescence stems validated the expressions of the three *IDD* genes detected by the GUS reporter (Figure 3D). Additionally, consistent with the previous finding that IDD members act as transcription factors [38], [39], an IDD14-GFP fusion protein in transgenic plants carrying *p35S::IDD14-GFP*, which recapitulated the phenotype of *idd14-1D*, was found to be localized in nuclei (Figure S2A, S2B).

### *IDD14*, *IDD15*, and *IDD16* Cooperatively Regulate Lateral Organ Morphogenesis and Gravitropic Responses

**Figure 4:**
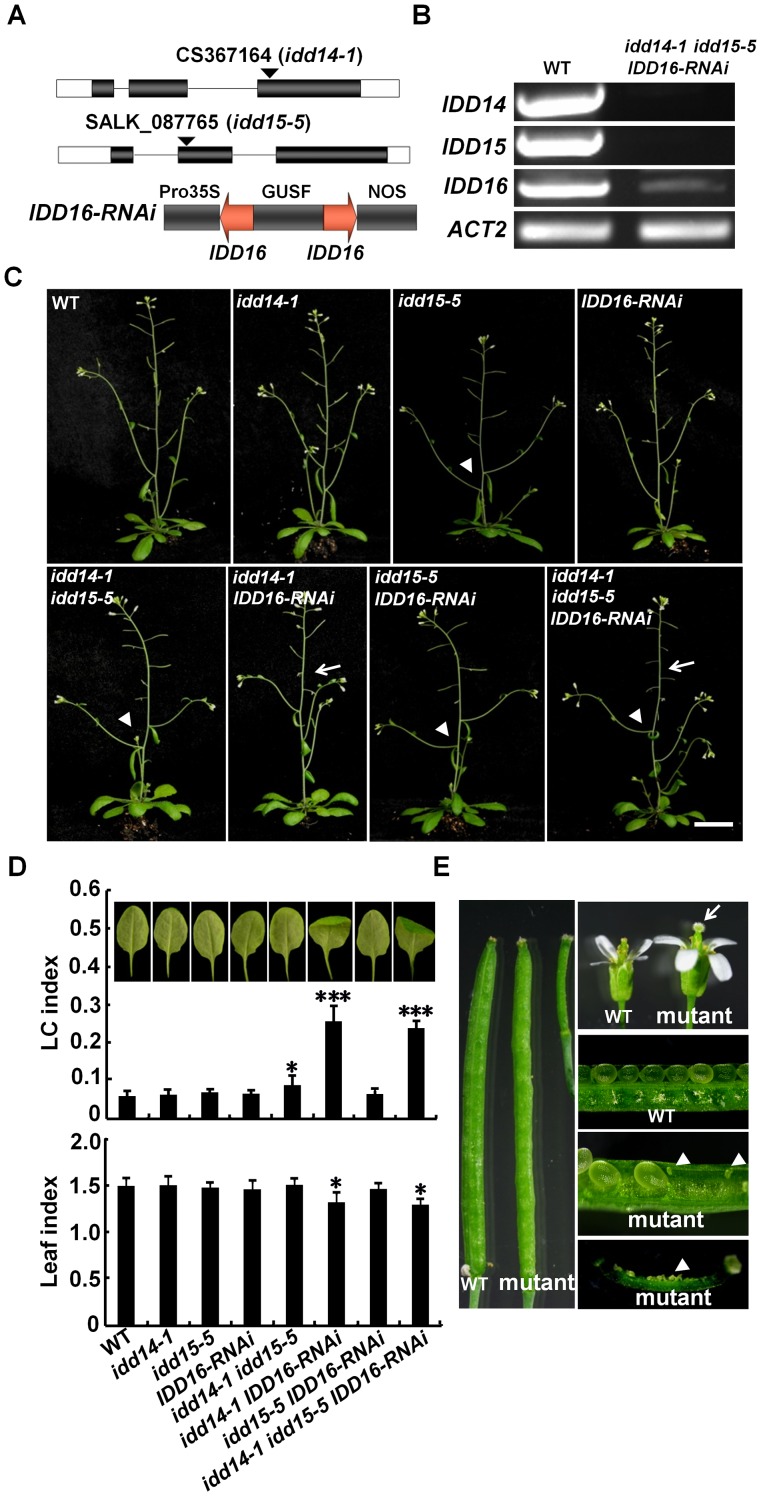
Pleiotropic organ phenotypes in loss-of-function *idd* mutants. (A) Schematic illustration of the *idd14-1*, *idd15-5*, and the *IDD16-RNAi* construct. A 248-bp specific *IDD16* cDNA fragment was used for construction of *p35S::IDD16-RNAi*. (B) Semi-quantitative RT-PCR analysis of *IDD* genes in WT and *idd* mutants. The transcripts of *IDD14*, *IDD15*, and *IDD16* in the *idd* triple mutant are shown as representatives. (C) 36-day-old plants of *idd* single, double, and triple mutants. Arrows indicate the infertile siliques. The arrowheads show the increased angles between the inflorescence stems and branches. The scale bar represents 2 cm. (D) Morphology of sixth leaves in 25-day-old *idd* mutants. The longitudinal curvature (LC) index and leaf index were determined from at least 10 leaves in each genotype. The data are shown as mean values ± one SD (Student's *t*-test, *P<0.05 and ***P<0.001). (E) Enlarged floral organs and infertile siliques in the *idd* triple mutant. The arrow indicates the stigma lacking pollen. The arrowheads show the unfertilized ovules.

To gain further insight into the functions of this *IDD* subfamily, we obtained the T-DNA insertion mutant *idd14-1* (CS367164) and *idd15-5* (Salk_087765) from the Arabidopsis Biological Resource Center (ABRC), in which the T-DNA is inserted in an exon of *IDD14* or *IDD15*. Semi-quantitative RT-PCR analyses indicated that the transcripts of *IDD14* or *IDD15* were undetectable in *idd14-1* or *idd15-5*, respectively (Figure 4A, 4B). As no *idd16* mutant is publically available, we generated *IDD16-RNAi* transgenic plants with an *IDD16*-specific cDNA fragment (Figure 4A). Semi-quantitative RT-PCR analysis indicated that *IDD16* transcripts were dramatically reduced in transgenic *IDD16-RNAi* plants (Figure 4B). We then generated double and triple mutants of the three *IDD* genes, which allowed us to closely examine their differential and redundant functions during plant growth and development.

We first examined the aerial organ morphogenesis in these *idd* mutants. As shown in Figure 4C and 4D, none of the *idd* single mutants exhibited any obvious organ phenotype. However, the leaves of *idd14-1 IDD16-RNAi* and *idd* triple mutants were not only downward-curled in the longitudinal direction, but were also more rotund when compared to WT leaves. Furthermore, *idd14-1 IDD16-RNAi* and the *idd* triple mutant had enlarged floral organs and infertile siliques. Careful examination showed that the infertile siliques resulted from the asynchronous elongation of stamen filaments and styles, and thus had poorly pollinated stigmas (Figure 4C, 4E). Manual pollination of styles in these mutants resulted in the development of normal siliques. In contrast to these dramatic phenotypes, the *idd15-5 IDD16-RNAi* plants had only slightly curled leaves (Figure 4C, 4D). These observations imply that *IDD14* and *IDD16* have redundant roles in directing leaf and floral organ morphogenesis.

**Figure 5:**
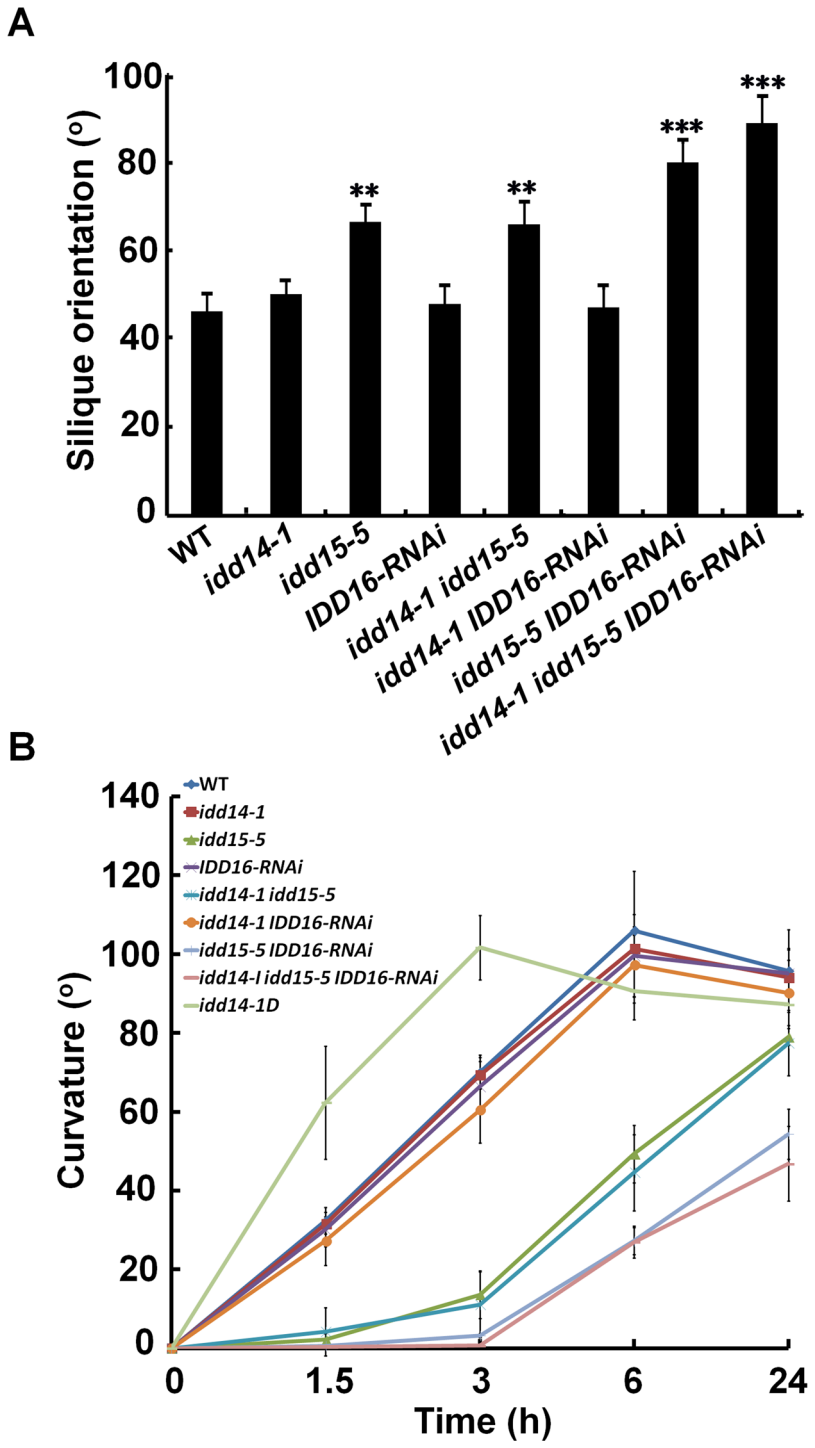
Silique orientation and gravitropic response in gain- and loss-of-function *idd* mutants. (A) Silique orientation in the *idd* single, double, and triple mutants. The angles between siliques and inflorescence stems were measured in 45-day-old WT and *idd* mutant plants. The data are shown as mean values ± one SD (Student's *t*-test, **P<0.01 and ***P<0.001). (B) Gravitropic responses of inflorescence stems in *idd14-1D* and loss-of-function *idd* mutants. The degree of curvature was determined from at least 18 inflorescence stems in each genotype after gravistimulation at the indicated times. The data are shown as mean values ± one SE.

Consistent with the previous finding that *idd15* displays increased angles between inflorescence stems and siliques [40], we noticed that the orientation angles of both branches and siliques were obviously increased in *idd15-5* (Figure 4C). This phenotype was further enhanced in the *idd15-5 IDD16-RNAi* and *idd* triple mutants, but not in *idd14-1 idd15-5* plants (Figure 4C, 5A), indicating that *IDD15* and *IDD16* act cooperatively to control silique and branch orientation. As *idd15* has a reduced gravitropic response in inflorescence stems [39]–[41], it is likely that the altered orientation of branches and siliques is related to the gravitropism defect in the *idd* mutants. To test this, we investigated the gravitropic responses in gain- and loss-of-function of *idd* plants. As expected, *idd15-5* inflorescence stems exhibited an obviously reduced gravitropic response, and this phenotype was greatly enhanced in *idd15-5 IDD16-RNAi* and the *idd* triple mutant (Figure 5B, S3A), demonstrating that *IDD15* and *IDD16* function coordinately in gravitropic responses. Interestingly, although *idd14-1* did not show any defect in gravitropic response, the inflorescence stems of *idd14-1D* were hypersensitive to gravistimulation (Figure 5B, S3A), indicating that ectopic expression of *IDD14* also influences gravitropic responses.

Surprisingly, although the *IDD* genes were found to be expressed in hypocotyls and roots, we did not observe any obvious phenotype in hypocotyls or roots of either *idd14-1D* or the *idd* triple mutant, including gravitropic response phenotypes (Figure S3B, S3C). The only aberration we observed was a slightly waved primary root phenotype in the *idd* triple mutant when grown vertically (Figure S3D). These findings suggest that the three *IDD*s may primarily function in lateral aerial organs.

### *IDD* Affects Auxin Accumulation by Modulating Auxin Homeostasis and Transport

**Figure 6:**
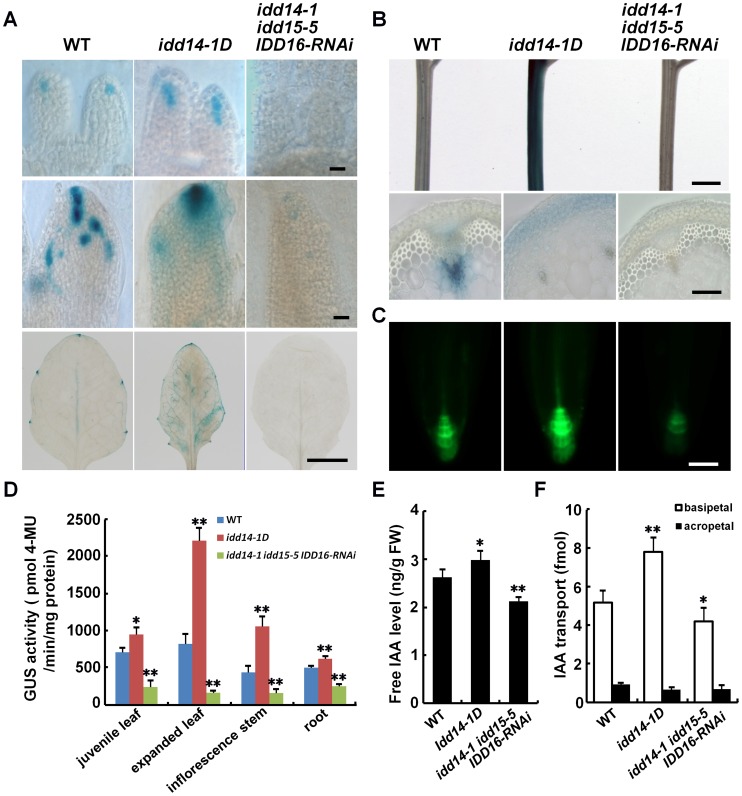
Altered auxin accumulation and transport in gain- and loss-of-function *idd* mutants. (A) Auxin accumulation assayed with the *DR5∶GUS* reporter in WT, *idd14-1D*, and *idd* triple mutant leaves. The leaves at 3, 5, and 15 days after initiation (from top to bottom panels) were subjected to GUS staining. The scale bars represent 50 µm in the top and middle panels and 5 mm in the bottom panel. (B) Expression of the *DR5∶GUS* reporter in inflorescence stems of WT, *idd14-1D*, and *idd* triple mutant plants. The scale bars represent 2 mm in the top panel and 100 µm in the bottom panel. (C) GFP fluorescence signals in the primary roots of WT, *idd14-1D*, and *idd* triple mutant carrying a *DR5∶GFP* reporter. The scale bar represents 50 µm. (D) Quantification of the GUS activity in the organs of the WT, *idd14-1D*, and *idd* triple mutant carrying a *DR5∶GUS* reporter. Data are from three biological replicates and shown as mean values ± one SD (Student's *t*-test, *P<0.05 and **P<0.01). (E) Endogenous free IAA levels in WT, *idd14-1D*, and *idd* triple mutant plants. Aerial organs of 15-day-old plants were used for measurement of free IAA levels. Data are from four biological replicates and are shown as mean values ± one SD (Student's *t*-test, *P<0.05 and **P<0.01). (F) Polar auxin transport capability of WT, *idd14-1D*, and *idd* triple mutant stems. The inflorescence stem segments of 5-week-old plants were used to determine the basipetal IAA transport efficiency and the background acropetal movement. Data are from four biological replicates and shown as mean values ± one SD (Student's *t*-test, *P<0.05 and **P<0.01).

Previous studies have suggested that *IDD14* and *IDD15* are involved in the regulation of starch metabolism [38], [40]. However, the narrow, epinastic leaves in gain-of-function *IDD* mutant or transgenic plants seem to resemble, to some extent, those observed in auxin overproduction mutants or transgenic plants, such as *yucD* or *p35S::YUC*s transgenic plants [6], [7], [9]. By contrast, the rotund and curly rosette leaves, abnormal floral phenotype, and gravitropism defect in the loss-of-function *idd* mutants are also documented in the mutants defective in auxin biosynthesis or transport [2], [9]–[11]. This led us speculate that auxin accumulation or signaling may also be involved in IDD-mediated organ development and/or gravitropic response. To test this, we used the *idd14-1D* and *idd* triple mutant plants as representatives of gain- and loss-of-function *idd* mutants for our further analysis. We first examined auxin accumulation in their leaf, inflorescence stem, and root, by monitoring the expression of *DR5∶GUS* or *DR5∶GFP*, a widely used auxin gradient reporter [50]. On day 3 after leaf initiation, an obvious GUS signal was observed in WT leaf distal tips. A stronger and spatially-expanded GUS signal was observed in *idd14-1D* leaf tips. No obvious GUS signal, and thus no localized auxin maxima, was observed in the leaf tips of *idd* triple mutant plants (Figure 6A). Similar differential GUS patterns were subsequently observed in 5-day-old expanding and 15-day-old expanded leaves (Figure 6A). In inflorescence stems, the GUS signal was mainly observed in vascular tissues in WT, whereas strong GUS staining was present in the cortex and endodermis tissues in *idd14-1D* and weak GUS expression without a tissue-specific pattern was observed in the *idd* triple mutant (Figure 6B). The increased or decreased auxin accumulation was also found in the meristem regions of primary roots in *idd14-1D* and the *idd* triple mutant, respectively (Figure 6C). Further quantification of the GUS activity in these organs confirmed the variations of DR5∶GUS signals observed in *idd14-1D* and the *idd* triple mutant (Figure 6D). This observation strongly suggests that alteration of *IDD* expression affects auxin accumulation in multiple organs.

Auxin homeostasis and transport are key factors that determine the accumulation of auxin in plant organs [1], [2], [4]. To assess the contributions of auxin homeostasis and transport to IDD-mediated auxin accumulation, we first quantified the endogenous free IAA levels in the *idd14-1D* and *idd* triple mutants. Consistent with enhanced or decreased expression of *DR5∶GUS* reporter observed, the endogenous IAA level was increased by about 13% in *idd14-1D* but decreased by approximately 19% in *idd* triple mutant plants as compared to that in WT (Figure 6E). This result implied that the *IDD* genes may affect auxin biosynthesis. We then measured the auxin transport capability of inflorescence stems in *idd14-1D* and *idd* triple mutant. As shown in Figure 6F, the basipetal IAA transport efficiency was increased by over 50% in *idd14-1D* stems but decreased about 18% in *idd* triple mutant stems when compared with that in WT stem, demonstrating that *IDD* also modulates the auxin transport process. To further examine whether auxin signaling is affected in gain- and loss-of-function *idd* mutants, we monitored the expression of the *DR5∶GUS* reporter, *IAA5*, and *IAA29* in response to exogenous IAA treatment in *idd14-1D* and the *idd* triple mutant, and observed that the expression of the *DR5∶GUS* reporter, *IAA5*, and *IAA29* was normally induced following application of IAA as that in WT plants (Figure S4A, S4B), suggesting that IDD has no effect on auxin perception or signaling. We also observed that the expression of *IDD14*, *IDD15*, and *IDD16* transcripts was not modulated by auxin treatment (Figure S4C). Taken together, our results strongly suggest that the three *IDD* genes are involved in the establishment of auxin gradients through the regulation of auxin biosynthesis and transport.

### IDD Directly Activates the Expression of *YUC5*, *TAA1*, and *PIN1*

**Figure 7:**
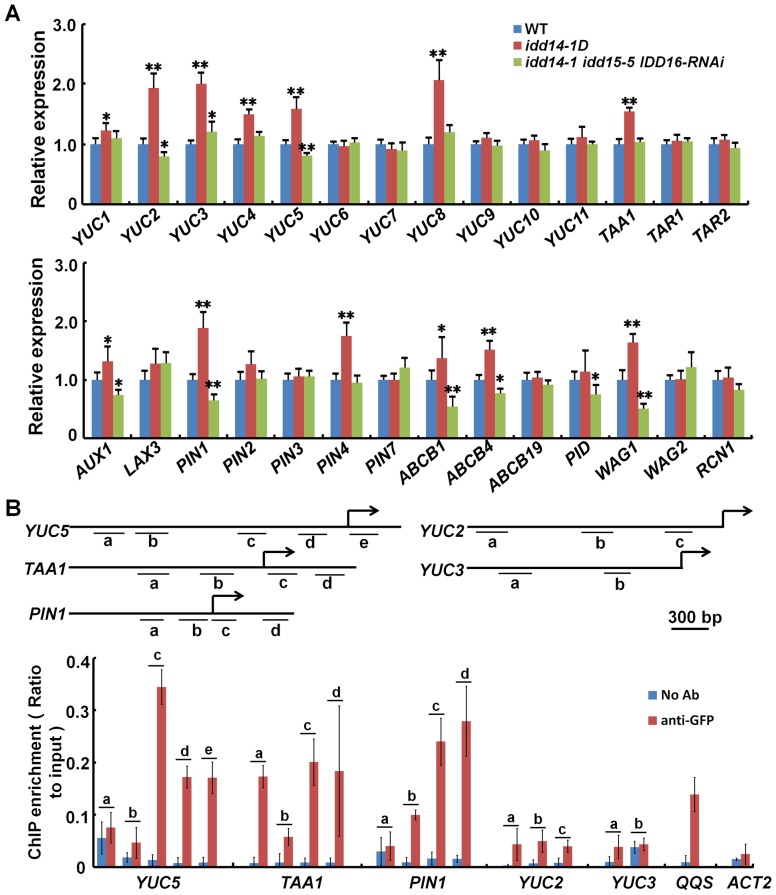
IDD Activates the expression of auxin biosynthetic and transport genes. (A) Relative expression levels of the genes involved in auxin biosynthesis (top panel) and transport (bottom panel) in WT, *idd14-1D*, and *idd* triple mutant plants. RNAs isolated from aerial organs of 4-week-old plants were subjected to qRT-PCR analysis, and the data are from three biological replicates and shown as mean values ± one SD (Student's *t*-test, *P<0.05 and **P<0.01). (B) ChIP assay performed with *p35S::IDD16-GFP* transgenic plants by anti-GFP antibody. The DNA fragments with a putative IDD-binding motif in the promoter and upstream regions of *YUC5*, *TAA1*, *PIN1*, *YUC2*, and *YUC3* are indicated as the letters a–e (top panel), and the enrichment values of their PCR products were quantified by qPCR (bottom panel). A reported IDD-targeted fragment in the *QQS* promoter and a fragment in the *ACT2* promoter were used as the positive and negative controls, respectively. The data are from three biological replicates and shown as mean values ± one SD.

To identify the genes downstream of IDD, we first carried out a real-time quantitative RT-PCR (qRT-PCR) analysis to examine the transcript abundances of genes known to function in auxin biosynthesis and transport in the *idd14-D* and *idd* triple mutants. Among the *YUC* and *TAA* family genes, the transcription of *YUC1*, *YUC2*, *YUC3*, *YUC4*, *YUC5*, *YUC8*, and *TAA1* was found to be elevated in *idd14-1D*, and the transcription levels of *YUC2* and *YUC5* were decreased in *idd* triple mutant, when compared to those in WT plants (Figure 7A). Among 14 genes related to auxin transport, the transcription of *AUX1*, *PIN1*, *ABCB1*, *ABCB4* and *WAG1* were found to be elevated in *idd14-1D* but reduced in the *idd* triple mutant compared to those in WT plants. *PIN4* and *PINOID* (*PID*) transcripts were only elevated in *idd14-1D* or deceased in the *idd* triple mutant (Figure 7A). We further investigated the expression of 11genes, which had the apparently altered expression in *idd14-1D* or the *idd* triple mutant (with a significance at P<0.01), in the transgenic plants overexpressing *IDD15* or *IDD16* and the *idd15-5 IDD16-RNAi* plants. As expected, their differential expressions in gain- and loss-of-function *IDD15* and *IDD16* plants were much similar to those observed in *idd14-1D* and the *idd* triple mutant (Figure S5A), demonstrating that these genes are also downstream of IDD15 and IDD16.

As the IDDs are transcription factors, we speculated that some of the genes differentially expressed in gain- and loss-of-function *idd* mutants might be directly targeted by IDD. We first identified the genes whose expression was rapidly induced by the activation of IDD, using the chemically-inducible *IDD14* transgenic plants. After the transgenic seedlings were treated with the inducer, *IDD14* was dramatically induced by 0.5 h, and the expression of *YUC5*, *TAA1*, and *PIN1* was obviously elevated within 2 h (Figure S5B), suggesting that three genes might be direct targets of IDDs. To examine whether IDD can directly bind to the promoter regions of *YUC5*, *TAA1*, or *PIN1*, we performed chromatin immunoprecipitation (ChIP) assays with both *p35S::IDD16-GFP* and *p35S::IDD14-GFP* transgenic plants. It has been reported that the maize ID1 and ID domain proteins could bind to a specific 11 bp DNA consensus motif, T-T-T-G-T-C-G/C-T/C-T/a-T/a-T [35]. As such, we targeted similar possible IDD-binding motifs in the promoter and/or upstream coding regions of *YUC5*, *TAA1*, *PIN1*, *YUC2*, and *YUC3*, and carried out the ChIP analysis (Figure 7B). As expected, we found that the three fragments containing a putative IDD-binding motif in *YUC5*, *TAA1*, or *PIN1* were greatly enriched by IDD16 after GFP immunoprecipitation, whereas no binding activity was detected in the promoter regions of *YUC*2 or *YUC3* (Figure 7B). Such enrichment was detected in a control DNA fragment of the *QUA-QUINE STARCH* (*QQS*) promoter, which was previously reported to be a target of IDD14 [38], but no enrichment was detectable in a control DNA fragment in the *ACTIN2* (*ACT2*) promoter which lacked the putative IDD-binding motif (Figure 7B). Likewise, similar enrichments of these DNA fragments were further confirmed by ChIP assayed with IDD14 protein (Figure S5C). In addition, we indeed visualized the enhanced or attenuated PIN1 accumulation in the roots of *idd14-1D* and *idd* triple mutant carrying a *pPIN1::PIN1-GFP* construct, respectively (Figure S6). These results illustrate that IDD can directly target *YUC5*, *TAA1*, and *PIN1*, to activate their expression.

### Alteration of Auxin Accumulation Suppresses Organ Morphogenesis in *idd* Mutants

**Figure 8:**
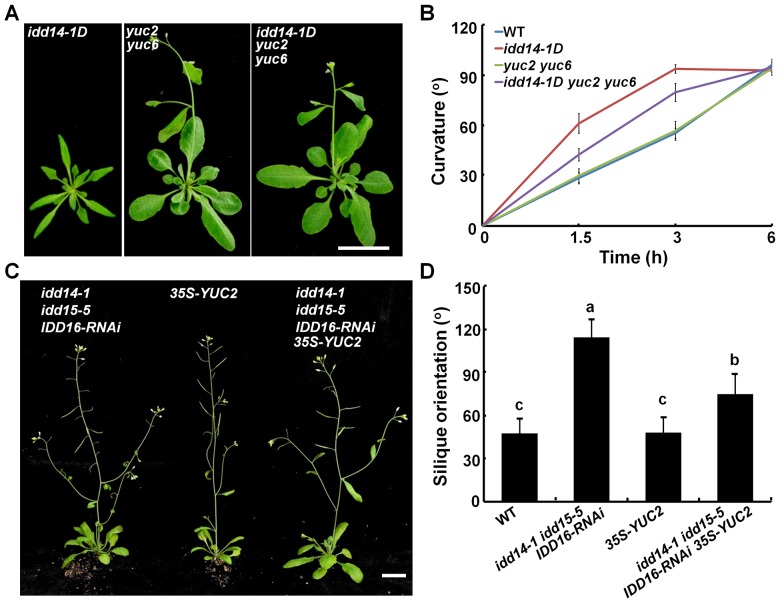
Genetic interaction of auxin biosynthesis and IDD-mediated organ morphogenesis and gravitropism. (A) Aerial organ morphology of 25-day-old *idd14-1D*, *yuc2 yuc6*, and *idd14-1D yuc2 yuc6* plants. The scale bar represents 2 cm. (B) Gravitropic responses of WT, *idd14-1D*, *yuc2 yuc6*, and *idd14-1D yuc2 yuc6* inflorescence stems. (C) 45-day-old plants of the *idd* triple mutant, *35S*-*YUC2*, and *idd* triple mutant carrying a *p35S::YUC2* construct. The scale bar represents 2 cm. (D) The silique orientation of WT, *idd* triple mutant, *35S*-*YUC2*, and *idd* triple mutant carrying a *p35S::YUC2* construct (one-way ANOVA test, P<0.05).

Since *idd14-1D* contains a high auxin level while the *idd* triple mutant has low endogenous auxin content, we attempted to genetically modify auxin biosynthesis to examine whether this could suppress or restore the phenotype observed in gain- and loss-of-function *idd* mutants. We first generated an *idd14-1D yuc2 yuc6* triple mutant through genetic crosses, and observed that loss-of-function *yuc2 yuc*6 completely suppressed the leaf phenotypes of *idd14-1D* (Figure 8A, S7), and partially attenuated the hypersensitivity of *idd14-1D* inflorescence stems to gravistimulation (Figure 8B). Further, when we overexpressed *YUC2* in the *idd* triple mutant, the ectopic expression of *YUC2* fully rescued the infertile silique defect (Figure 8C), but only partially restored the silique orientation in the *idd* triple mutant (Figure 8D). These results provide further evidence that auxin biosynthesis is genetically downstream of this IDD subfamily.

As the amyloplast movement in endodermal cells of *idd15* stem has been reported to be defective under gravistimulation [39]–[41], we also investigated whether ectopic expression of *YUC2* has an effect on amyloplast movement in endodermal cells of the *idd* triple mutant stems. As shown in Figure S8, the retarded amyloplast movement in endodermal cells of the *idd* triple mutant stems under gravistimulation was not rescued by ectopically expressed *YUC2* (Figure S8), suggesting that altered auxin accumulation does not affect IDD-mediated amyloplast responsiveness to gravistimulation.

## Discussion

### The Distinct IDD Subfamily Is Critical for Organ Development and Plant Architecture Formation

The IDD family has been defined as a plant-specific transcription factor family [35], [51], and previous characterizations of a few IDD members have indicated that IDDs are involved in the regulation of transition to flowering and starch metabolism [36]–[38]. IDD14, IDD15, and IDD16 belong to a subfamily that is distinct from the other IDD members in *Arabidopsis*, rice, and maize [34]. The mutation of *IDD15* in *Arabidopsis* and rice reduces gravitropic response in inflorescence stems [39]–[41], [48]. However, because the loss-of-function *idd14* mutant does not have obvious organ phenotype, and *IDD16* has not yet been characterized, the functions of this subfamily are still largely unknown.

In this study, we characterized both gain- and loss-of-function mutants of this IDD subfamily, and discovered that these three *IDD* genes function redundantly and cooperatively in regulating organ morphogenesis and gravitropic responses. Gain-of-function of each *IDD* led to a small, narrow, and down-curled leaf phenotype. Although *idd* single mutants did not have obvious organ morphological phenotypes (except the reduced gravitropic response observed in *idd15*), our further characterization of the *idd* double and triple mutants clearly demonstrates that *IDD14* and *IDD16* act redundantly to regulate the morphology of aerial organs and affect fertility, while *IDD15* and *IDD16* cooperatively control the gravitropic responses and plant architecture.

Such redundant and cooperative roles of the three *IDD* genes in organ morphogenesis and gravitropism are consistent with their differential and overlapping expression patterns in particular organs. *IDD14* and *IDD16*, but not *IDD15*, were expressed in juvenile leaves, whereas *IDD15* and *IDD16*, but not *IDD14*, were highly expressed in inflorescence stems. The three *IDD* genes were all expressed in floral organs, and the abnormal flower phenotype in *idd14-1 IDD16-RNAi* was enhanced in the *idd* triple mutant. Interestingly, although the *IDD* genes were also expressed in hypocotyls and roots, we could not observe obvious changes in their morphology or gravitropic response in either gain- or loss-of-function *idd* mutants, except the slightly waved roots in *idd* triple mutant seedlings. This may be attributable to decreased expression of *WAG1*, a gene that is an indirect target of IDD and has been identified as a suppressor of root waving (Figure 7A) [52].

### IDD14, IDD15, and IDD16 Regulate Auxin Gradients by Promoting Auxin Biosynthesis and Transport

Previous studies have shown that IDD15 and IDD14 are involved in the regulation of starch metabolism [38], [40]. Here, with a combination of phenotypic, genetic, and molecular approaches, we demonstrated that IDD14, IDD15, and IDD16 modulate auxin accumulation by affecting auxin biosynthesis and transport, thereby modifying organ morphogenesis and architecture formation. First, the organ phenotype in both gain- and loss-of-function *idd* mutants appears to be related to altered auxin homeostasis and distribution. For example, the narrow, epinastic leaves in the plants overexpressing *IDD* are similar to those in auxin overproduction mutants or transgenic plants, such as *yucca* (*yuc1D*), *yucca6-1D*, and *p35S::YUC*s transgenic plants [6], [7], [9], whereas the rotund and curly rosette leaves and abnormal floral phenotype in the loss-of-function *idd* mutants are similar to those observed in the mutants defective in auxin biosynthesis or transport [9], [53]. Disruption of *IDD* genes also influenced the silique and branch angles, root waving, and gravitropism of inflorescence stems, which have been well documented to be related to polar auxin transport [2], [54]–[57]. Second, expression analyses using *DR5∶GUS* and *DR5∶GFP* reporter clearly indicated that gain- or loss-of-function of *IDD*s enhanced or reduced auxin gradients, which was further confirmed by the increased or decreased endogenous auxin content and transport ability. Third, the expression of several genes involved in auxin biosynthesis and transport were altered in both gain- and loss-of-function *idd* mutants, and the IDD proteins could directly bind to the promoter regions of *YUC5*, *TAA1*, and *PIN1* to activate their expression. In addition, genetic manipulation of auxin biosynthesis could fully or partially restore the pleiotropic phenotypes in *idd14-1D* or the *idd* triple mutant. These results demonstrate that IDD indeed modulates auxin gradients by promoting auxin biosynthesis and transport.

### IDD-Mediated Auxin Gradients and Starch Metabolism May Coordinate Gravitropic Response

The gravitropic response in plants requires a coordination of three sequential processes: gravity perception, signal transduction, and asymmetric growth response [58]. It is widely believed that the starch-filled amyloplasts (statoliths) within specific gravi-sensing cells (statocytes) perceive gravity stimulation [59], [60]. Some other molecules, such as InsP_3_ and Ca^2+^ have been found to be involved in gravity signaling [60]. A large body of evidence indicates that auxin plays a key role in gravitropic signaling and asymmetric organ growth, and that it may possibly be involved in gravi-sensing [2], [60]. For example, many mutants related to auxin biosynthesis and especially transport such as *taa1*, *aux1*, *pin1*, and *pin2*, exhibit a defect in gravitropic responses [2], [11], [61]. Other mutants with altered silique or branch architecture, such as *plethora* (*plt*) in *Arabidopsis* and *lazy1* (*la1*) in rice, also show altered auxin accumulation or transport within their organs [54], [56], [62].

Among the three IDD members we characterized, IDD15 and its rice ortholog, LPA1, have been previously reported to affect the gravitropic response by altering amyloplast sedimentation in the endodermis [39]–[41], [48]. Recently, IDD14 was also found to mediate starch degradation by directly activating the expression of *QQS* [38], suggesting that IDD14 also participates in the regulation of starch metabolism. Our detailed characterization of gain- and loss-of-function of three *IDD*s provides substantial evidence that IDD-mediated auxin biosynthesis and transport contribute to the organ morphogenesis and also, to some extent, gravitropic responses, because the genetic manipulation of auxin biosynthesis does not alter the responsiveness of amyloplast to gravistimulation but partially restores the gravity sensitivity or defect in gain- or loss-of-function *idd* mutants. Therefore, it is likely that IDD-mediated auxin accumulation and starch metabolism coordinately control the gravitropic responses. The IDD-regulated starch metabolism might be primarily involved in gravi-sensing while the IDD-regulated auxin gradient may be primarily involved in signaling and responses.

## Materials and Methods

### Plant Materials and Growth Conditions

The *Arabidopsis thaliana* accession Col-0 was used in this study. *idd14-1D* was isolated from a population generated by T-DNA activation-tagging mutagenesis. *idd14-1* (CS367164) and *idd15-5* (SALK_087765) were obtained from ABRC. All seeds were sterilized and geminated on 1/2 MS medium after vernalization for 2 days at 4°C, and the plants were grown in a culture room or growth chamber at 22±1°C with an illumination intensity of 80–90 µmol m^−2^ s^−1^ and a 16-h light/8-h dark photoperiod, as described previously [63].

### Leaf Curvature and Gravitropism Assays

Leaf curvature was determined as described previously [64], [65]. Briefly, the transverse curvature (TC) index was defined as TC = 1- cw/pw, where cw and pw are the curved width and the pressed width of leaves, respectively. The longitudinal curvature (LC) index was defined as LC = 1- cl/pl, where cl and pl are the curved length and the pressed length of leaves, respectively. To quantify the pressed width or length of leaves, the sixth leaf of 25-day-old plants was dissected transversely or longitudinally on a desk, and then the extreme distances between the margins of the leaf were measured before and after pressing.

To quantify the gravitropic responses of roots and hypocotyls, seedlings were grown vertically for 4 days and then turned horizontally, and curvature angles were measured [58]. For quantification of the gravitropic responses of inflorescence stems, 32-day-old plants with inflorescence stems of approximately 4–8 cm were gravistimulated by rotating them 90° in darkness, and stem curvatures were measured as the angles between the growing direction of apex and horizontal base line [39], [58].

### Genotyping of T-DNA Mutants

The T-DNA flanking sequence of *idd14-1D* was amplified by TAIL-PCR [66]. Three primers, P1, P2, and pSK-LB2 were used for co-segregation analysis. P1 and P2 were located in the *Arabidopsis* genome flanking the T-DNA insertion site, and pSK-LB2 was a primer corresponding to the left border of the T-DNA sequence. Similarly, *idd14-1* and *idd15-5* were genotyped with corresponding primers (Table S2), and *yuc2* and *yuc6* were genotyped according to the methods from a previous study [9].

### Plasmid Construction and Plant Transformation

To generate *p35S::IDD*s and *p35S::anti-IDD14* transgenic plants, the *IDD* coding sequences were amplified by RT-PCR and ligated into the pGEM-T-Easy vector (Promega, USA), and then verified by sequencing. The resulting plasmids were digested with *Eco*RI and cloned into pVIP96 [67]. The *IDD14* cDNA was also cloned into pER8 to generate a chemically inducible *IDD14* construct. To investigate the tissue-specific expression of *IDD14*, *IDD15*, and *IDD16*, approximately 2-kb promoter fragments of *IDD* genes were amplified from genomic DNA and then fused with the β-glucuronidase (*GUS*) gene into pBI101. To generate the *p35S::IDD16-GFP* and *p35S::IDD14-GFP* constructs, an *IDD16* or *IDD14* coding sequence lacking a stop codon was amplified and then cloned in frame into pMDC83 (Invitrogen, USA). To generate the *p35S::IDD16-RNAi* construct, a specific *IDD16* cDNA fragment was amplified and ligated inversely into pBluescript SK-GUSF [68], and then an *Xba*I-*Bgl*II digested fragment was sub-cloned into pVIP96. A *YUC2* cDNA fragment was amplified by RT-PCR and cloned into pVIP96myc to generate the *p35S::YUC2* construct. All the primers used are listed in Table S2.

All constructs were introduced into *Arabidopsis* by *Agrobacterium tumefaciens*–mediated transformation via the floral dip as described previously [69]. At least 20 independent lines harboring a single T-DNA insertion from each construct were generated, and 4–5 independent lines of T3 homozygous plants were used for detailed characterization.

### Gene Expression Analysis

Total RNA was isolated using a guanidine thiocyanate extraction buffer [70]. For semi-quantitative RT-PCR or qRT-PCR analysis, cDNA was synthesized from 1 µg of total RNA using SuperScript III Reverse Transcriptase (Invitrogen, USA). qRT-PCR was performed with a Rotor-Gene 3000 thermocycler (Corbett Research, Australian) with a SYBR *Premix Ex Taq* II kit (Takara, Japan). The relative expression level for each gene was normalized to the *ACTIN2* and the data were collected from three biological replicates, as described previously [49].

The histochemical GUS assay was carried out according to previously described protocol [71]. The GUS activities were quantified by monitoring cleavage of the β-glucuronidase substrate 4-methylumbelliferyl β-D-glucuronide (MUG), as described [71].

For RNA *in situ* hybridization, a specific cDNA region of *IDD14*, *IDD15*, or *IDD16* was transcribed *in vitro* to generate sense and antisense probes using the Digoxigenin RNA labeling kit (Roche, Switzerland). The WT inflorescence stems were fixed and embedded in paraffin (Sigma-Aldrich, USA), and then sectioned to a 10 µm thickness. RNA *in situ* hybridization was performed according to a previously described method [54].

### Free IAA Measurement

Aerial organs from 15-day-old plants of WT, *idd14-1D*, and *idd* triple mutant plants were used for measurement of free IAA content. The extraction, purification, and analysis of free IAA by gas chromatography-mass spectrometry was performed according to the methods described by Edlund *et al.* [72], except that an Agilent GC and a LECO Pegasus TOF mass spectrometer was used, with separation using a DB-5ht column (Agilent, USA) [63].

### Auxin Transport Assays

Auxin transport in inflorescence stems was measured according to the methods of a previously published protocol [73]. 25-mm inflorescence segments were cut from 5-week-old plants, and the segments were submerged inversely into an auxin transport buffer (100 nM ^3^H-IAA, 0.05% MES, pH 5.5–5.7) in a 0.5-ml micro-centrifuge tube. Control experiments were performed by submerging the base of inflorescence stems to measure acropetal IAA movement. After 12 h, 5-mm stem segments were dissected from the non-submerged ends and used to quantify the radiolabeled auxin using a scintillation counter.

### Chromatin Immunoprecipitation (ChIP) Assays

ChIP assays were performed as described previously [74]. Briefly, 2 g of *p35S::IDD16-GFP* or *p35S::IDD14-GFP* transgenic plants grown on 1/2 MS plates for 16 days was harvested, and then submerged in 1% formaldehyde to crosslink the DNA with DNA-binding proteins. The chromatin pellets were extracted and sheared by sonication. 5 µl anti-GFP antibodies (Abcam, UK) were used to immunoprecipitate the DNA-IDD16 or DNA-IDD14 complexes. DNA was released with proteinase K and then purified. The enrichment of DNA fragments was determined by quantitative PCR with the primers listed in Table S2.

### Amyloplast Staining

Plants were grown in soil until the primary inflorescence stems bolted to a height of 4–9 cm, and then gravistimulated by rotating them upside down [40]. 1-cm-long inflorescence stems below the apex were fixed, embedded in paraffin, and sectioned to a 10-µm thickness. A periodic acid-Schiff kit (Sigma-Aldrich, USA) was used for amyloplast staining, according to the manufacturer's instructions.

## Supporting Information

Figure S1 IDD14, IDD15, and IDD16 belong to a distinct IDD subfamily. (A) Phylogeny of the *Arabidopsis* IDD family. A neighbor-joining tree of 16 IDD members was generated using CLUSTAL W based on the amino acid sequences of IDD proteins. Number of generations = 1000. (B) Transcriptional analysis of *IDD* genes in transgenic plants overexpressing *IDD14*, *IDD15*, or *IDD16*, respectively. Two independent lines of each construct were assayed by semi-quantitative RT-PCR and the expression of At1g68140 was determined in *35S-IDD14* plants. The *GLYCERALDEHYDE-3-PHOSPHATE DEHYDROGENASE C SUBUNIT* (*GAPC*) gene was used as an internal control.
(TIF)

Figure S2 IDD14-GFP protein is localized in nucleus. (A) Phenotype of 25-day-old *p35S::IDD14-GFP* transgenic plant. The scale bar represents 1 cm. (B) Nuclear localization of the IDD14-GFP protein in transgenic plants. GFP fluorescence, DAPI staining, overlaid and bright fields (BF) are shown from left to right. The scale bar represents 10 µm.
(TIF)

Figure S3 Gravitropic response and root morphology of gain- and loss-of-function *idd* mutants. (A) Gravitropic responses of inflorescence stem in *idd* and *idd14-1D* plants. 32-day-old plants were gravistimulated by rotation by 90° in the dark for 0, 3, or 24 h. The scale bar represents 2 cm. (B–C) Kinetics of the gravitropic response of the hypocotyls (B) and primary roots (C) in WT, *idd14-1D*, and *idd* triple mutant plants. At least 20 seedlings from each genotype were used. The data are shown as mean values ± one SE. (D) Morphology of primary roots of WT, *idd14-1D*, and *idd* triple mutant plants. Seedlings grown vertically for 6 days were photographed. Note that the primary roots in the *idd* triple mutant showed a slightly waving phenotype. The scale bar represents 0.5 cm.
(TIF)

Figure S4 Auxin responses of *idd* mutants and *IDD* genes. (A) Expression of *DR5∶GUS* in WT, *idd14-1D*, and *idd* triple mutant seedlings treated with/without auxin. The 5-day-old seedlings were treated with various concentrations of IAA for 6 h and subjected to GUS staining assays. The scale bar represents 2 mm. (B) Transcripts of *IAA5* and *IAA29* in WT, *idd14-1D*, and *idd* triple mutant before and after auxin treatment. (C) Transcript levels of *IDD14*, *IDD15*, and *IDD16* in WT plants before and after auxin treatment. Semi-quantitative RT-PCR was performed with the RNAs isolated from 10-day-old seedlings treated with 1 µM IAA for the time durations indicated. *GAPC* was used as an internal control.
(TIF)

Figure S5 Identification of IDD-regulated genes involved in auxin biosynthesis and transport. (A) Relative expression levels of the genes involved in auxin biosynthesis and transport in WT, *35S-IDD15*, *35S-IDD16*, and *idd15-5 IDD16-RNAi* mutant plants. RNAs isolated from aerial organs of 3-week-old plants were subjected to qRT-PCR analysis, and data are from three biological replicates and shown as mean values ± one SD (Student's *t*-test, *P<0.05 and **P<0.01). (B) Expression analyses of IDD-regulated genes in transgenic plants carrying an inducible *IDD14* construct. 15-day-old transgenic plants were transferred into a liquid medium containing DMSO or 10 µM β-estradiol for the indicated time durations, and subjected to RNA isolation and qRT-PCR analysis. Data are from three biological replicates and shown as mean values ± one SD. Note that *YUC5*, *TAA1*, and *PIN1* are rapidly induced by the activation of IDD14. (C) ChIP assay performed with *p35S::IDD14-GFP* transgenic plants by anti-GFP antibody. The DNA fragments with a possible IDD-binding motif in the promoter and upstream regions of *YUC5*, *TAA1*, *PIN1*, *YUC2*, and *YUC3* (a–e) were assayed by ChIP, and the enrichments of their qPCR products are shown as mean values ± one SD from three biological replicates. An IDD-targeted fragment in the *QQS* promoter and a fragment in the *ACT2* promoter were used as the positive and negative controls, respectively.
(TIF)

Figure S6 PIN1 accumulation in gain- and loss-of-function *idd* mutants. GFP fluorescent signals in primary roots of WT, *idd14-1D*, and *idd* triple mutant plants containing a *pPIN1::PIN1-GFP* construct. GFP fluorescence, bright fields (BF), and overlaid images are shown from left to right. The scale bar represents 50 µm.
(TIF)

Figure S7 Suppression of *idd14-1D* phenotypes by *yuc2 yuc6*. (A–C) The blade areas (A), transverse curvature (TC) index (B), and leaf index (C) of WT, *idd14-1D*, *yuc2 yuc6*, and *idd14-1D yuc2 yuc6* leaves. At least 10 sixth leaves from each genotype were used for determination of the leaf area, TC index, and leaf index, respectively. Data are shown as mean values ± one SD (one-way ANOVA test, P<0.05).
(TIF)

Figure S8 Alteration of auxin biosynthesis does not affect amyloplast sedimentation in *idd* mutants. Plants were gravistimulated by turning upside down for 0 or 20 min, and longitudinal sections of inflorescence stems were prepared and then stained with a periodic acid-Schiff kit. Arrowheads indicate the retarded movement of amyloplasts in the endodermal cells of the triple *idd* mutant and triple *idd* mutant carrying a *p35S::YUC2* construct. The scale bar represents 10 µm.
(TIF)

Table S1 Phenotypic characterization of *cuf1-D* plants.
(DOC)

Table S2 Primers used in this study.
(DOC)

## References

[1] Zhao Y (2010) Auxin biosynthesis and its role in plant development. Annu Rev Plant Biol 61: 49–64. 20192736 10.1146/annurev-arplant-042809-112308 PMC3070418

[2] Petrášek J, Friml J (2009) Auxin transport routes in plant development. Development 136: 2675–2688. 19633168 10.1242/dev.030353

[3] Hayashi K (2012) The interaction and integration of auxin signaling components. Plant Cell Physiol 53: 965–975. 22433459 10.1093/pcp/pcs035

[4] Woodward AW, Bartel B (2005) Auxin: regulation, action, and interaction. Ann Bot 95: 707–735. 15749753 10.1093/aob/mci083 PMC4246732

[5] Vanneste S, Friml J (2009) Auxin: a trigger for change in plant development. Cell 136: 1005–1016. 19303845 10.1016/j.cell.2009.03.001

[6] Zhao Y, Christensen SK, Fankhauser C, Cashman JR, Cohen JD, et al (2001) A role for flavin monooxygenase-like enzymes in auxin biosynthesis. Science 291: 306–309. 11209081 10.1126/science.291.5502.306

[7] Kim JI, Sharkhuu A, Jin JB, Li P, Jeong JC, et al (2007) *yucca6*, a dominant mutation in *Arabidopsis*, affects auxin accumulation and auxin-related phenotypes. Plant Physiol 145: 722–735. 17885085 10.1104/pp.107.104935 PMC2048792

[8] Woodward C, Bemis SM, Hill EJ, Sawa S, Koshiba T, et al (2005) Interaction of auxin and ERECTA in elaborating *Arabidopsis* inflorescence architecture revealed by the activation tagging of a new member of the YUCCA family putative flavin monooxygenases. Plant Physiol 139: 192–203. 16126863 10.1104/pp.105.063495 PMC1203369

[9] Cheng Y, Dai X, Zhao Y (2006) Auxin biosynthesis by the YUCCA flavin monooxygenases controls the formation of floral organs and vascular tissues in *Arabidopsis* . Genes Dev 20: 1790–1799. 16818609 10.1101/gad.1415106 PMC1522075

[10] Cheng Y, Dai X, Zhao Y (2007) Auxin synthesized by the YUCCA flavin monooxygenases is essential for embryogenesis and leaf formation in *Arabidopsis* . Plant Cell 19: 2430–2439. 17704214 10.1105/tpc.107.053009 PMC2002601

[11] Stepanova AN, Robertson-Hoyt J, Yun J, Benavente LM, Xie DY, et al (2008) TAA1-mediated auxin biosynthesis is essential for hormone crosstalk and plant development. Cell 133: 177–191. 18394997 10.1016/j.cell.2008.01.047

[12] Tao Y, Ferrer JL, Ljung K, Pojer F, Hong F, et al (2008) Rapid synthesis of auxin via a new tryptophan-dependent pathway is required for shade avoidance in plants. Cell 133: 164–176. 18394996 10.1016/j.cell.2008.01.049 PMC2442466

[13] Yamada M, Greenham K, Prigge MJ, Jensen PJ, Estelle M (2009) The *TRANSPORT INHIBITOR RESPONSE2* gene is required for auxin synthesis and diverse aspects of plant development. Plant Physiol 151: 168–179. 19625638 10.1104/pp.109.138859 PMC2735986

[14] Mashiguchi K, Tanaka K, Sakai T, Sugawara S, Kawaide H, et al (2011) The main auxin biosynthesis pathway in *Arabidopsis* . Proc Natl Acad Sci USA 108: 18512–18517. 22025724 10.1073/pnas.1108434108 PMC3215075

[15] Won C, Shen X, Mashiguchi K, Zheng Z, Dai X, et al (2011) Conversion of tryptophan to indole-3-acetic acid by TRYPTOPHAN AMINOTRANSFERASES OF ARABIDOPSIS and YUCCAs in *Arabidopsis* . Proc Natl Acad Sci USA 108: 18518–18523. 22025721 10.1073/pnas.1108436108 PMC3215067

[16] Zhao Y (2012) Auxin biosynthesis: a simple two-step pathway converts tryptophan to indole-3-acetic acid in plants. Mol Plant 5: 334–338. 22155950 10.1093/mp/ssr104 PMC3309920

[17] Sohlberg JJ, Myrenås M, Kuusk S, Lagercrantz U, Kowalczyk M, et al (2006) STY1 regulates auxin homeostasis and affects apical-basal patterning of the *Arabidopsis* gynoecium. Plant J 47: 112–123. 16740145 10.1111/j.1365-313X.2006.02775.x

[18] Trigueros M, Navarrete-Gómez M, Sato S, Christensen SK, Pelaz S, et al (2009) The *NGATHA* genes direct style development in the *Arabidopsis* gynoecium. Plant Cell 21: 1394–1409. 19435937 10.1105/tpc.109.065508 PMC2700528

[19] Stone SL, Braybrook SA, Paula SL, Kwong LW, Meuser J, et al (2008) *Arabidopsis* LEAFY COTYLEDON2 induces maturation traits and auxin activity: Implications for somatic embryogenesis. Proc Natl Acad Sci USA 105: 3151–3156. 18287041 10.1073/pnas.0712364105 PMC2268600

[20] Sun J, Qi L, Li Y, Chu J, Li C (2012) PIF4-mediated activation of *YUCCA8* expression integrates temperature into the auxin pathway in regulating *Arabidopsis* hypocotyl growth. PLoS Genet 8: e1002594. 22479194 10.1371/journal.pgen.1002594 PMC3315464

[21] Li LC, Qin GJ, Tsuge T, Hou XH, Ding MY, et al (2008) *SPOROCYTELESS* modulates *YUCCA* expression to regulate the development of lateral organs in *Arabidopsis* . New Phytol 179: 751–764. 18557819 10.1111/j.1469-8137.2008.02514.x

[22] Bennett MJ, Marchant A, Green HG, May ST, Ward SP, et al (1996) *Arabidopsis AUX1* gene: a permease-like regulator of root gravitropism. Science 273: 948–950. 8688077 10.1126/science.273.5277.948

[23] Petrášek J, Mravec J, Bouchard R, Blakeslee JJ, Abas M, et al (2006) PIN proteins perform a rate-limiting function in cellular auxin efflux. Science 312: 914–918. 16601150 10.1126/science.1123542

[24] Swarup K, Benková E, Swarup R, Casimiro I, Péret B, et al (2008) The auxin influx carrier LAX3 promotes lateral root emergence. Nat Cell Biol 10: 946–954. 18622388 10.1038/ncb1754

[25] Bainbridge K, Guyomarc'h S, Bayer E, Swarup R, Bennett M, et al (2008) Auxin influx carriers stabilize phyllotactic patterning. Genes Dev 22: 810–823. 18347099 10.1101/gad.462608 PMC2275433

[26] Jones AR, Kramer EM, Knox K, Swarup R, Bennett MJ, et al (2009) Auxin transport through non-hair cells sustains root-hair development. Nat Cell Biol 11: 78–84. 19079245 10.1038/ncb1815 PMC2635559

[27] Marchant A, Bhalerao R, Casimiro I, Eklöf J, Casero PJ, et al (2002) AUX1 promotes lateral root formation by facilitating indole-3-acetic acid distribution between sink and source tissues in the *Arabidopsis* seedling. Plant Cell 14: 589–597. 11910006 10.1105/tpc.010354 PMC150581

[28] Stone BB, Stowe-Evans EL, Harper RM, Celaya RB, Ljung K, et al (2008) Disruptions in AUX1-dependent auxin influx alter hypocotyl phototropism in *Arabidopsis* . Mol Plant 1: 129–144. 20031920 10.1093/mp/ssm013

[29] Benková E, Michniewicz M, Sauer M, Teichmann T, Seifertová D, et al (2003) Local, efflux-dependent auxin gradients as a common module for plant organ formation. Cell 115: 591–602. 14651850 10.1016/s0092-8674(03)00924-3

[30] Chen R, Hilson P, Sedbrook J, Rosen E, Caspar T, et al (1998) The *Arabidopsis thaliana AGRAVITROPIC 1* gene encodes a component of the polar-auxin-transport efflux carrier. Proc Natl Acad Sci USA 95: 15112–15117. 9844024 10.1073/pnas.95.25.15112 PMC24584

[31] Friml J, Vieten A, Sauer M, Weijers D, Schwarz H, et al (2003) Efflux-dependent auxin gradients establish the apical-basal axis of *Arabidopsis* . Nature 426: 147–153. 14614497 10.1038/nature02085

[32] Mravec J, Kubeš M, Bielach A, Gaykova V, Petrášek J, et al (2008) Interaction of PIN and PGP transport mechanisms in auxin distribution-dependent development. Development 135: 3345–3354. 18787070 10.1242/dev.021071

[33] Scarpella E, Marcos D, Friml J, Berleth T (2006) Control of leaf vascular patterning by polar auxin transport. Genes Dev 20: 1015–1027. 16618807 10.1101/gad.1402406 PMC1472298

[34] Colasanti J, Tremblay R, Wong AY, Coneva V, Kozaki A, et al (2006) The maize *INDETERMINATE1* flowering time regulator defines a highly conserved zinc finger protein family in higher plants. BMC Genomics 7: 158. 16784536 10.1186/1471-2164-7-158 PMC1586020

[35] Kozaki A, Hake S, Colasanti J (2004) The maize ID1 flowering time regulator is a zinc finger protein with novel DNA binding properties. Nucleic Acids Res 32: 1710–1720. 15020707 10.1093/nar/gkh337 PMC390334

[36] Colasanti J, Yuan Z, Sundaresan V (1998) The *INDETERMINATE1* gene encodes a zinc finger protein and regulates a leaf-generated signal required for the transition to flowering in maize. Cell 93: 593–603. 9604934 10.1016/s0092-8674(00)81188-5

[37] Seo PJ, Ryu J, Kang SK, Park CM (2011) Modulation of sugar metabolism by an INDETERMINATE DOMAIN transcription factor contributes to photoperiodic flowering in *Arabidopsis* . Plant J 65: 418–429. 21265895 10.1111/j.1365-313X.2010.04432.x

[38] Seo PJ, Kim MJ, Ryu JY, Jeong EY, Park CM (2011) Two splice variants of the IDD14 transcription factor competitively form nonfunctional heterodimers which may regulate starch metabolism. Nat Commun 2: 303. 21556057 10.1038/ncomms1303

[39] Morita MT, Sakaguchi K, Kiyose S, Taira K, Kato T, et al (2006) A C2H2-type zinc finger protein, SGR5, is involved in early events of gravitropism in *Arabidopsis* inflorescence stems. Plant J 47: 619–628. 16813575 10.1111/j.1365-313X.2006.02807.x

[40] Tanimoto M, Tremblay R, Colasanti J (2008) Altered gravitropic response, amyloplast sedimentation and circumnutation in the *Arabidopsis shoot gravitropism 5* mutant are associated with reduced starch levels. Plant Mol Biol 67: 57–69. 18259878 10.1007/s11103-008-9301-0

[41] Yamauchi Y, Fukaki H, Fujisawa H, Tasaka M (1997) Mutations in the *SGR4*, *SGR5* and *SGR6* loci of *Arabidopsis thaliana* alter the shoot gravitropism. Plant Cell Physiol 38: 530–535. 9210330 10.1093/oxfordjournals.pcp.a029201

[42] Levesque MP, Vernoux T, Busch W, Cui H, Wang JY, et al (2006) Whole-genome analysis of the SHORT-ROOT developmental pathway in *Arabidopsis* . PLoS Biol 4: e143. 16640459 10.1371/journal.pbio.0040143 PMC1450008

[43] Welch D, Hassan H, Blilou I, Immink R, Heidstra R, et al (2007) *Arabidopsis* JACKDAW and MAGPIE zinc finger proteins delimit asymmetric cell division and stabilize tissue boundaries by restricting SHORT-ROOT action. Genes Dev 21: 2196–2204. 17785527 10.1101/gad.440307 PMC1950858

[44] Feurtado JA, Huang D, Wicki-Stordeur L, Hemstock LE, Potentier MS, et al (2011) The *Arabidopsis* C2H2 zinc finger INDETERMINATE DOMAIN1/ENHYDROUS promotes the transition to germination by regulating light and hormonal signaling during seed maturation. Plant Cell 23: 1772–1794. 21571950 10.1105/tpc.111.085134 PMC3123948

[45] Matsubara K, Yamanouchi U, Wang ZX, Minobe Y, Izawa T, et al (2008) *Ehd2*, a rice ortholog of the maize *INDETERMINATE1* gene, promotes flowering by up-regulating *Ehd1* . Plant Physiol 148: 1425–1435. 18790997 10.1104/pp.108.125542 PMC2577255

[46] Park SJ, Kim SL, Lee S, Je BI, Piao HL, et al (2008) Rice *Indeterminate 1* (*OsId1*) is necessary for the expression of *Ehd1* (*Early heading date 1*) regardless of photoperiod. Plant J 56: 1018–1029. 18774969 10.1111/j.1365-313X.2008.03667.x

[47] Wu C, You C, Li C, Long T, Chen G, et al (2008) *RID1*, encoding a Cys2/His2-type zinc finger transcription factor, acts as a master switch from vegetative to floral development in rice. Proc Natl Acad Sci USA 105: 12915–12920. 18725639 10.1073/pnas.0806019105 PMC2529042

[48] Wu X, Tang D, Li M, Wang K, Cheng Z (2013) Loose Plant Architecture 1, an INDETERMINATE domain protein involved in shoot gravitropism, regulates plant architecture in rice. Plant Physiol 161: 317–329. 23124325 10.1104/pp.112.208496 PMC3532263

[49] Hu Z, Qin Z, Wang M, Xu C, Feng G, et al (2010) The *Arabidopsis* SMO2, a homologue of yeast TRM112, modulates progression of cell division during organ growth. Plant J 61: 600–610. 19929876 10.1111/j.1365-313X.2009.04085.x

[50] Ulmasov T, Murfett J, Hagen G, Guilfoyle TJ (1997) Aux/IAA proteins repress expression of reporter genes containing natural and highly active synthetic auxin response elements. Plant Cell 9: 1963–1971. 9401121 10.1105/tpc.9.11.1963 PMC157050

[51] Englbrecht CC, Schoof H, Böhm S (2004) Conservation, diversification and expansion of C2H2 zinc finger proteins in the *Arabidopsis thaliana* genome. BMC Genomics 5: 39. 15236668 10.1186/1471-2164-5-39 PMC481060

[52] Santner AA, Watson JC (2006) The WAG1 and WAG2 protein kinases negatively regulate root waving in *Arabidopsis* . Plant J 45: 752–764. 16460509 10.1111/j.1365-313X.2005.02641.x

[53] Blakeslee JJ, Bandyopadhyay A, Lee OR, Mravec J, Titapiwatanakun B, et al (2007) Interactions among PIN-FORMED and P-glycoprotein auxin transporters in *Arabidopsis* . Plant Cell 19: 131–147. 17237354 10.1105/tpc.106.040782 PMC1820964

[54] Li P, Wang Y, Qian Q, Fu Z, Wang M, et al (2007) *LAZY1* controls rice shoot gravitropism through regulating polar auxin transport. Cell Res 17: 402–410. 17468779 10.1038/cr.2007.38

[55] Yoshihara T, Iino M (2007) Identification of the gravitropism-related rice gene *LAZY1* and elucidation of LAZY1-dependent and -independent gravity signaling pathways. Plant Cell Physiol 48: 678–688. 17412736 10.1093/pcp/pcm042

[56] Prasad K, Grigg SP, Barkoulas M, Yadav RK, Sanchez-Perez GF, et al (2011) *Arabidopsis* PLETHORA transcription factors control phyllotaxis. Curr Biol 21: 1123–1128. 21700457 10.1016/j.cub.2011.05.009

[57] Oliva M, Dunand C (2007) Waving and skewing: how gravity and the surface of growth media affect root development in *Arabidopsis* . New Phytol 176: 37–43. 17692076 10.1111/j.1469-8137.2007.02184.x

[58] Fukaki H, Fujisawa H, Tasaka M (1996) *SGR1*, *SGR2*, *SGR3*: novel genetic loci involved in shoot gravitropism in *Arabidopsis thaliana* . Plant Physiol 110: 945–955. 8819871 10.1104/pp.110.3.945 PMC157794

[59] Hashiguchi Y, Tasaka M, Morita MT (2013) Mechanism of higher plant gravity sensing. Am J Bot 100: 91–100. 23115136 10.3732/ajb.1200315

[60] Baldwin KL, Strohm AK, Masson PH (2013) Gravity sensing and signal transduction in vascular plant primary roots. Am J Bot 100: 126–142. 23048015 10.3732/ajb.1200318

[61] Haga K, Sakai T (2012) PIN auxin efflux carriers are necessary for pulse-induced but not continuous light-induced phototropism in *Arabidopsis* . Plant Physiol 160: 763–776. 22843667 10.1104/pp.112.202432 PMC3461554

[62] Pinon V, Prasad K, Grigg SP, Sanchez-Perez GF, Scheres B (2012) Local auxin biosynthesis regulation by PLETHORA transcription factors controls phyllotaxis in *Arabidopsis* . Proc Natl Acad Sci USA 110: 1107–1112. 23277580 10.1073/pnas.1213497110 PMC3549086

[63] Jing Y, Cui D, Bao F, Hu Z, Qin Z, et al (2009) Tryptophan deficiency affects organ growth by retarding cell expansion in *Arabidopsis* . Plant J 57: 511–521. 18980661 10.1111/j.1365-313X.2008.03706.x

[64] Nath U, Crawford BC, Carpenter R, Coen E (2003) Genetic control of surface curvature. Science 299: 1404–1407. 12610308 10.1126/science.1079354

[65] Wu F, Yu L, Cao W, Mao Y, Liu Z, et al (2007) The N-terminal double-stranded RNA binding domains of *Arabidopsis* HYPONASTIC LEAVES1 are sufficient for pre-microRNA processing. Plant Cell 19: 914–925. 17337628 10.1105/tpc.106.048637 PMC1867364

[66] Liu YG, Mitsukawa N, Oosumi T, Whittier RF (1995) Efficient isolation and mapping of *Arabidopsis thaliana* T-DNA insert junctions by thermal asymmetric interlaced PCR. Plant J 8: 457–463. 7550382 10.1046/j.1365-313x.1995.08030457.x

[67] Hu Y, Xie Q, Chua NH (2003) The *Arabidopsis* auxin-inducible gene *ARGOS* controls lateral organ size. Plant Cell 15: 1951–1961. 12953103 10.1105/tpc.013557 PMC181323

[68] Qin G, Gu H, Zhao Y, Ma Z, Shi G, et al (2005) An indole-3-acetic acid carboxyl methyltransferase regulates *Arabidopsis* leaf development. Plant Cell 17: 2693–2704. 16169896 10.1105/tpc.105.034959 PMC1242266

[69] Clough SJ, Bent AF (1998) Floral dip: a simplified method for *Agrobacterium*-mediated transformation of *Arabidopsis thaliana* . Plant J 16: 735–743. 10069079 10.1046/j.1365-313x.1998.00343.x

[70] Hu Y, Bao F, Li J (2000) Promotive effect of brassinosteroids on cell division involves a distinct CycD3-induction pathway in *Arabidopsis* . Plant J 24: 693–701. 11123807 10.1046/j.1365-313x.2000.00915.x

[71] Jefferson RA, Kavanagh TA, Bevan MW (1987) GUS fusions: beta-glucuronidase as a sensitive and versatile gene fusion marker in higher plants. EMBO J 6: 3901–3907. 3327686 10.1002/j.1460-2075.1987.tb02730.x PMC553867

[72] Edlund A, Eklöf S, Sundberg B, Moritz T, Sandberg G (1995) A microscale technique for gas chromatography-mass spectrometry measurements of picogram amounts of indole-3-acetic acid in plant tissues. Plant Physiol 108: 1043–1047. 12228526 10.1104/pp.108.3.1043 PMC157455

[73] Lewis DR, Muday GK (2009) Measurement of auxin transport in *Arabidopsis thaliana* . Nat Protoc 4: 437–451. 19282849 10.1038/nprot.2009.1

[74] Gendrel AV, Lippman Z, Martienssen R, Colot V (2005) Profiling histone modification patterns in plants using genomic tiling microarrays. Nat Methods 2: 213–218. 16163802 10.1038/nmeth0305-213

