# Supplementary figures and images for: The Arabidopsis IDD14, IDD15, and IDD16 Cooperatively Regulate Lateral Organ Morphogenesis and Gravitropism by Promoting Auxin Biosynthesis and Transport
Source: PLoS Genet. 2013 Sep 5;9(9):e1003759. doi: 10.1371/journal.pgen.1003759 (PMC3764202; doi:10.1371/journal.pgen.1003759)

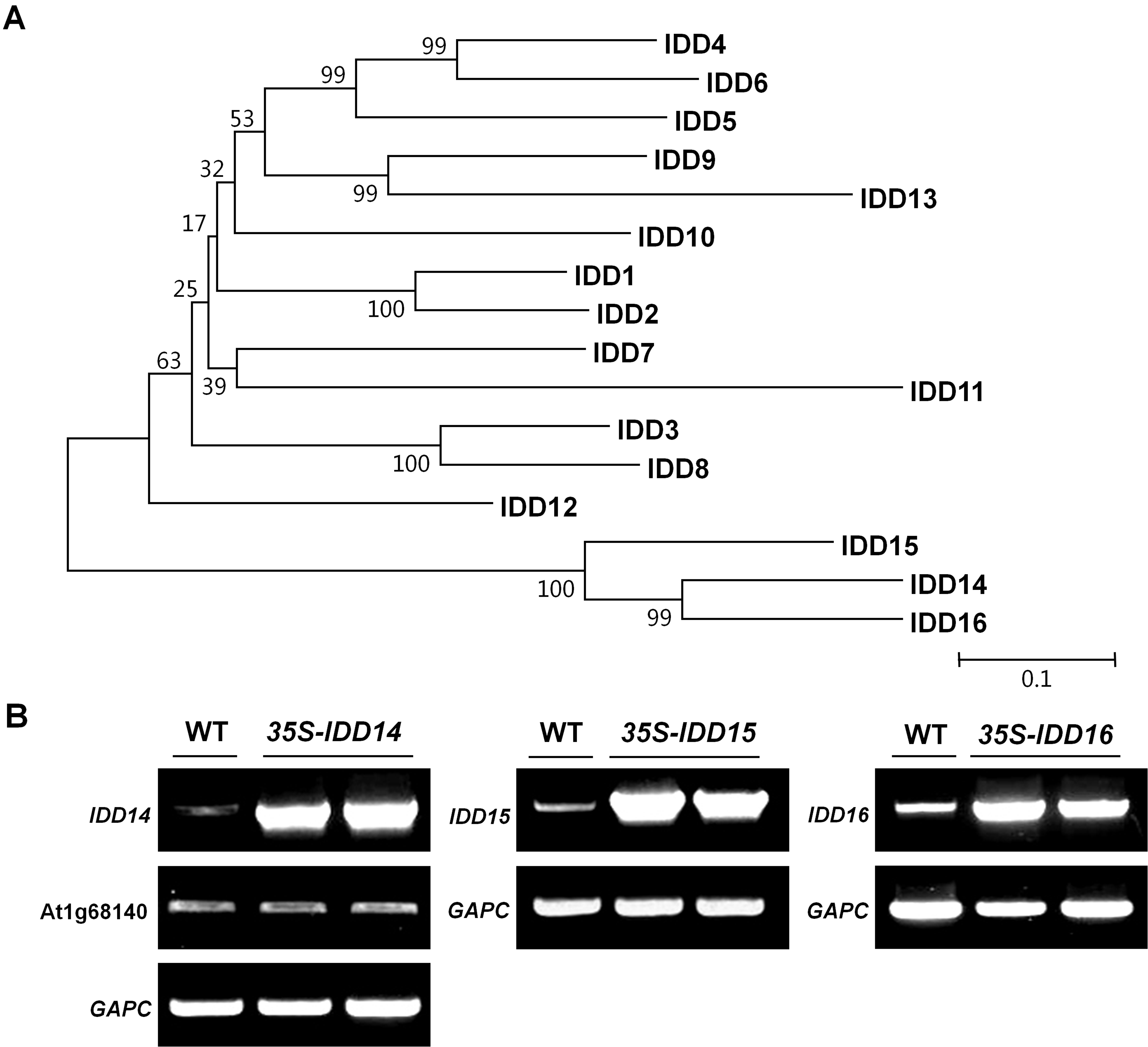

Supplement: Figure S1 — IDD14, IDD15, and IDD16 belong to a distinct IDD subfamily. (A) Phylogeny of the Arabidopsis IDD family. A neighbor-joining tree of 16 IDD members was generated using CLUSTAL W based on the amino acid sequences of IDD proteins. Number of generations = 1000. (B) Transcriptional analysis of IDD genes in transgenic plants overexpressing IDD14, IDD15, or IDD16, respectively. Two independent lines of each construct were assayed by semi-quantitative RT-PCR and the expression of At1g68140 was determined in 35S-IDD14 plants. The GLYCERALDEHYDE-3-PHOSPHATE DEHYDROGENASE C SUBUNIT (GAPC) gene was used as an internal control. (TIF) [file pgen.1003759.s001.tif]

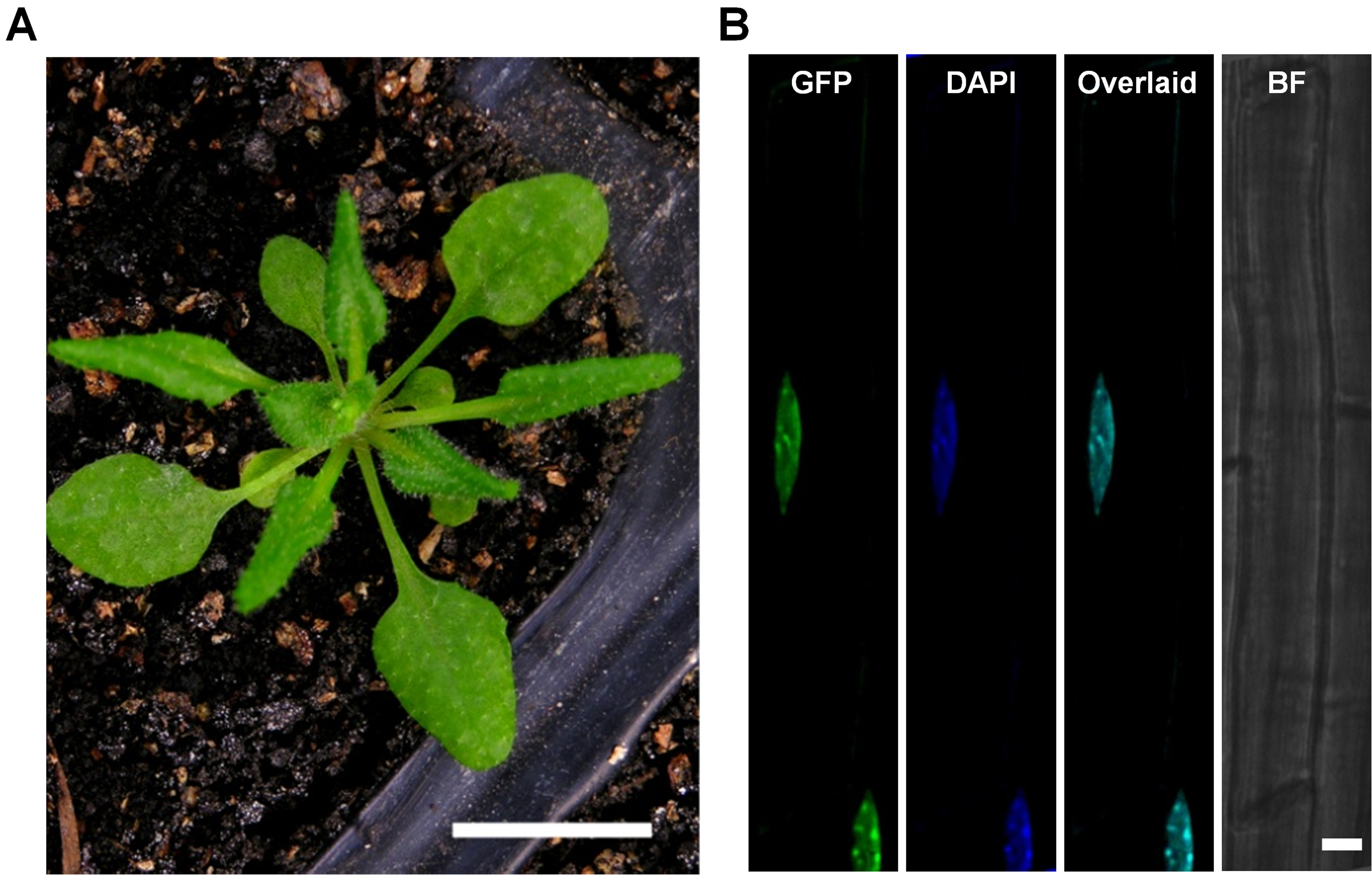

Supplement: Figure S2 — IDD14-GFP protein is localized in nucleus. (A) Phenotype of 25-day-old p35S::IDD14-GFP transgenic plant. The scale bar represents 1 cm. (B) Nuclear localization of the IDD14-GFP protein in transgenic plants. GFP fluorescence, DAPI staining, overlaid and bright fields (BF) are shown from left to right. The scale bar represents 10 µm. (TIF) [file pgen.1003759.s002.tif]

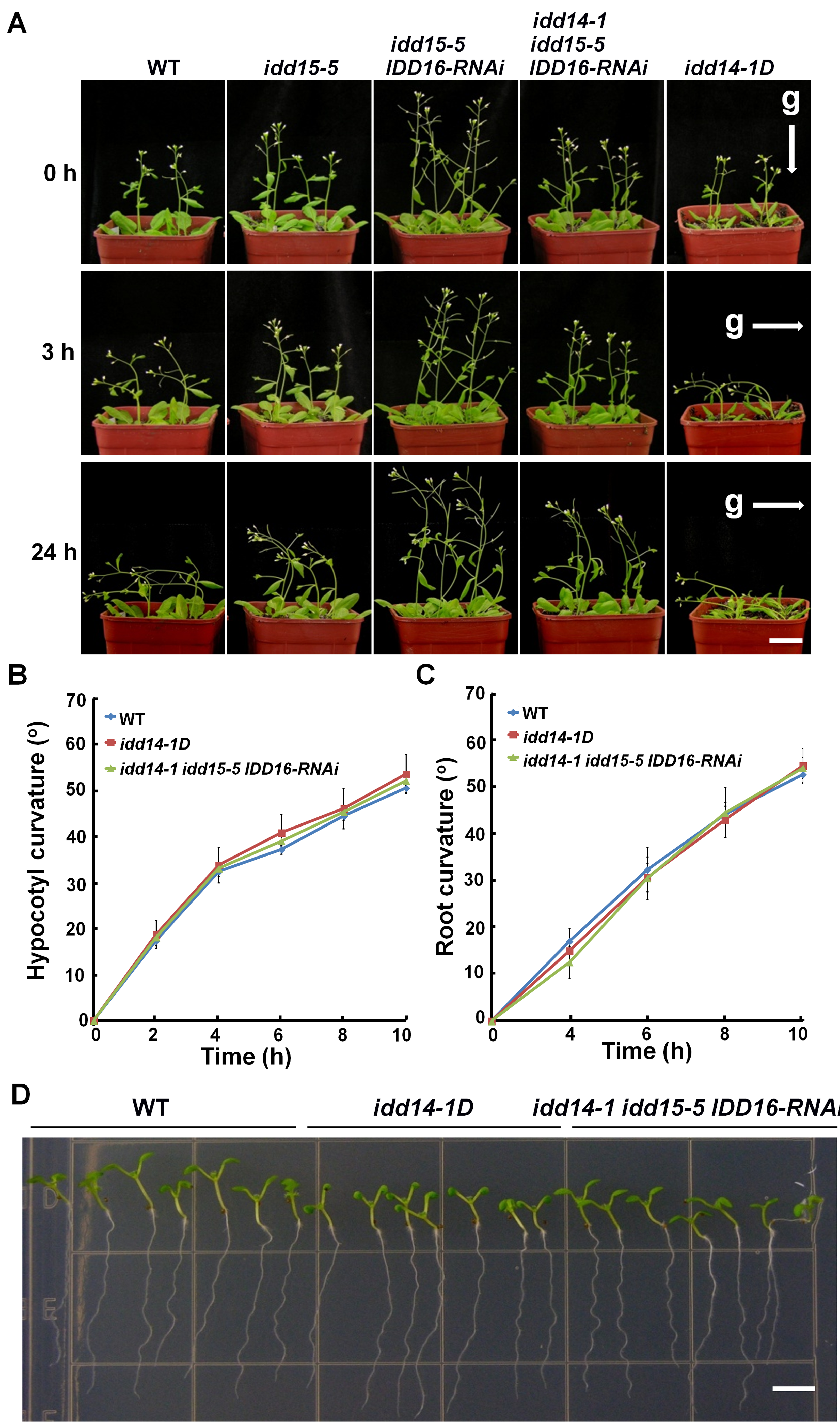

Supplement: Figure S3 — Gravitropic response and root morphology of gain- and loss-of-function idd mutants. (A) Gravitropic responses of inflorescence stem in idd and idd14-1D plants. 32-day-old plants were gravistimulated by rotation by 90° in the dark for 0, 3, or 24 h. The scale bar represents 2 cm. (B–C) Kinetics of the gravitropic response of the hypocotyls (B) and primary roots (C) in WT, idd14-1D, and idd triple mutant plants. At least 20 seedlings from each genotype were used. The data are shown as mean values ± one SE. (D) Morphology of primary roots of WT, idd14-1D, and idd triple mutant plants. Seedlings grown vertically for 6 days were photographed. Note that the primary roots in the idd triple mutant showed a slightly waving phenotype. The scale bar represents 0.5 cm. (TIF) [file pgen.1003759.s003.tif]

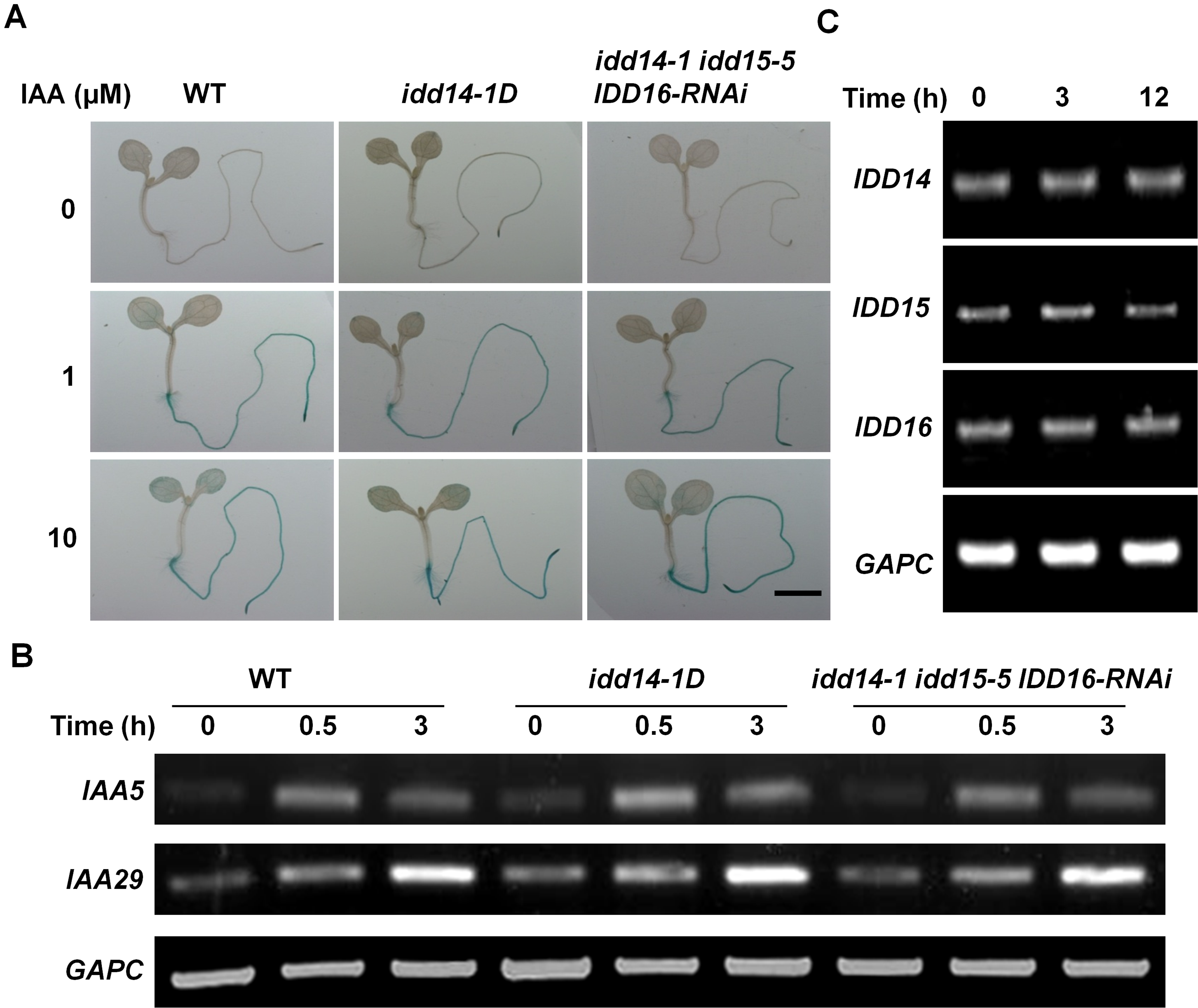

Supplement: Figure S4 — Auxin responses of idd mutants and IDD genes. (A) Expression of DR5∶GUS in WT, idd14-1D, and idd triple mutant seedlings treated with/without auxin. The 5-day-old seedlings were treated with various concentrations of IAA for 6 h and subjected to GUS staining assays. The scale bar represents 2 mm. (B) Transcripts of IAA5 and IAA29 in WT, idd14-1D, and idd triple mutant before and after auxin treatment. (C) Transcript levels of IDD14, IDD15, and IDD16 in WT plants before and after auxin treatment. Semi-quantitative RT-PCR was performed with the RNAs isolated from 10-day-old seedlings treated with 1 µM IAA for the time durations indicated. GAPC was used as an internal control. (TIF) [file pgen.1003759.s004.tif]

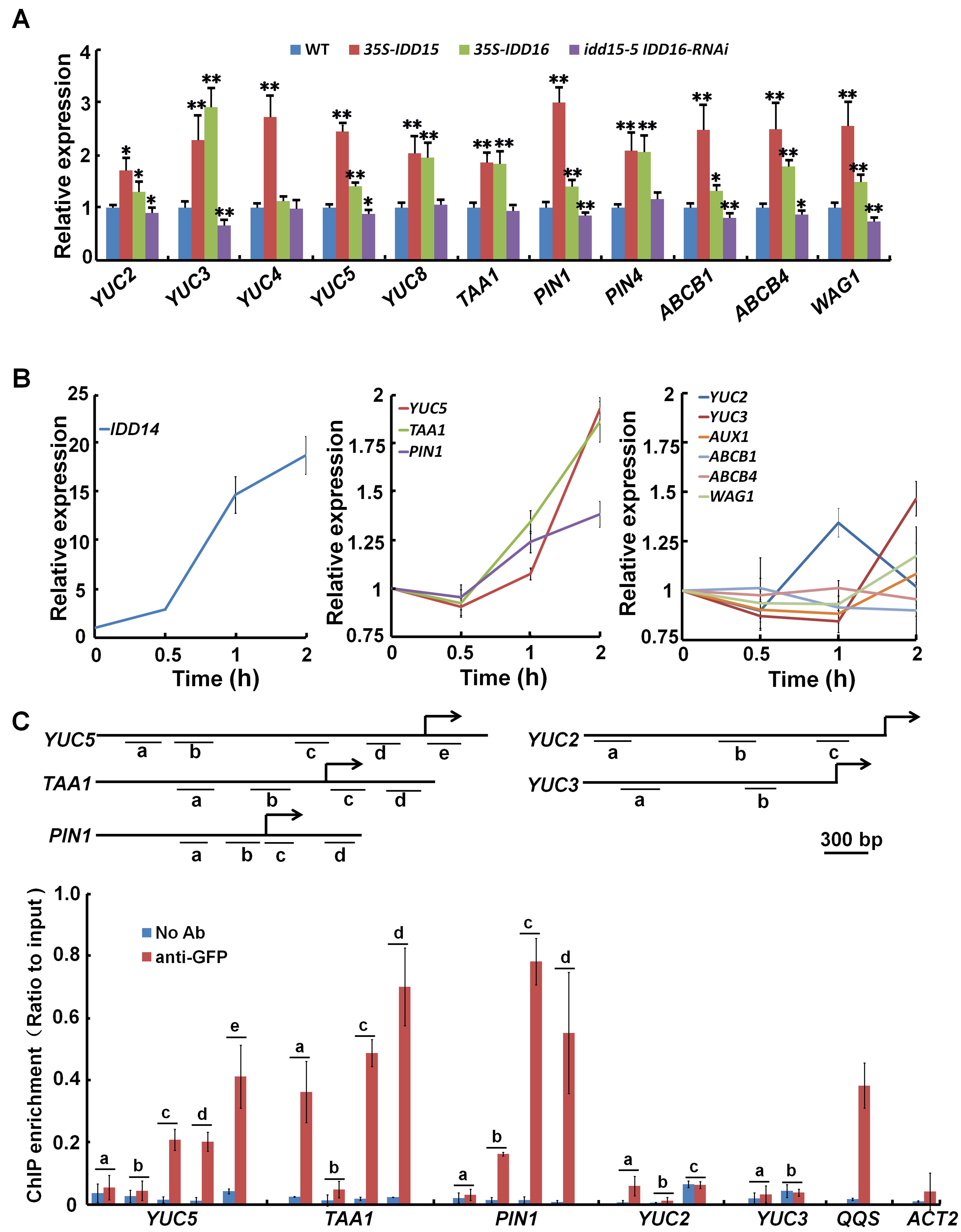

Supplement: Figure S5 — Identification of IDD-regulated genes involved in auxin biosynthesis and transport. (A) Relative expression levels of the genes involved in auxin biosynthesis and transport in WT, 35S-IDD15, 35S-IDD16, and idd15-5 IDD16-RNAi mutant plants. RNAs isolated from aerial organs of 3-week-old plants were subjected to qRT-PCR analysis, and data are from three biological replicates and shown as mean values ± one SD (Student's t-test, *P<0.05 and **P<0.01). (B) Expression analyses of IDD-regulated genes in transgenic plants carrying an inducible IDD14 construct. 15-day-old transgenic plants were transferred into a liquid medium containing DMSO or 10 µM β-estradiol for the indicated time durations, and subjected to RNA isolation and qRT-PCR analysis. Data are from three biological replicates and shown as mean values ± one SD. Note that YUC5, TAA1, and PIN1 are rapidly induced by the activation of IDD14. (C) ChIP assay performed with p35S::IDD14-GFP transgenic plants by anti-GFP antibody. The DNA fragments with a possible IDD-binding motif in the promoter and upstream regions of YUC5, TAA1, PIN1, YUC2, and YUC3 (a–e) were assayed by ChIP, and the enrichments of their qPCR products are shown as mean values ± one SD from three biological replicates. An IDD-targeted fragment in the QQS promoter and a fragment in the ACT2 promoter were used as the positive and negative controls, respectively. (TIF) [file pgen.1003759.s005.tif]

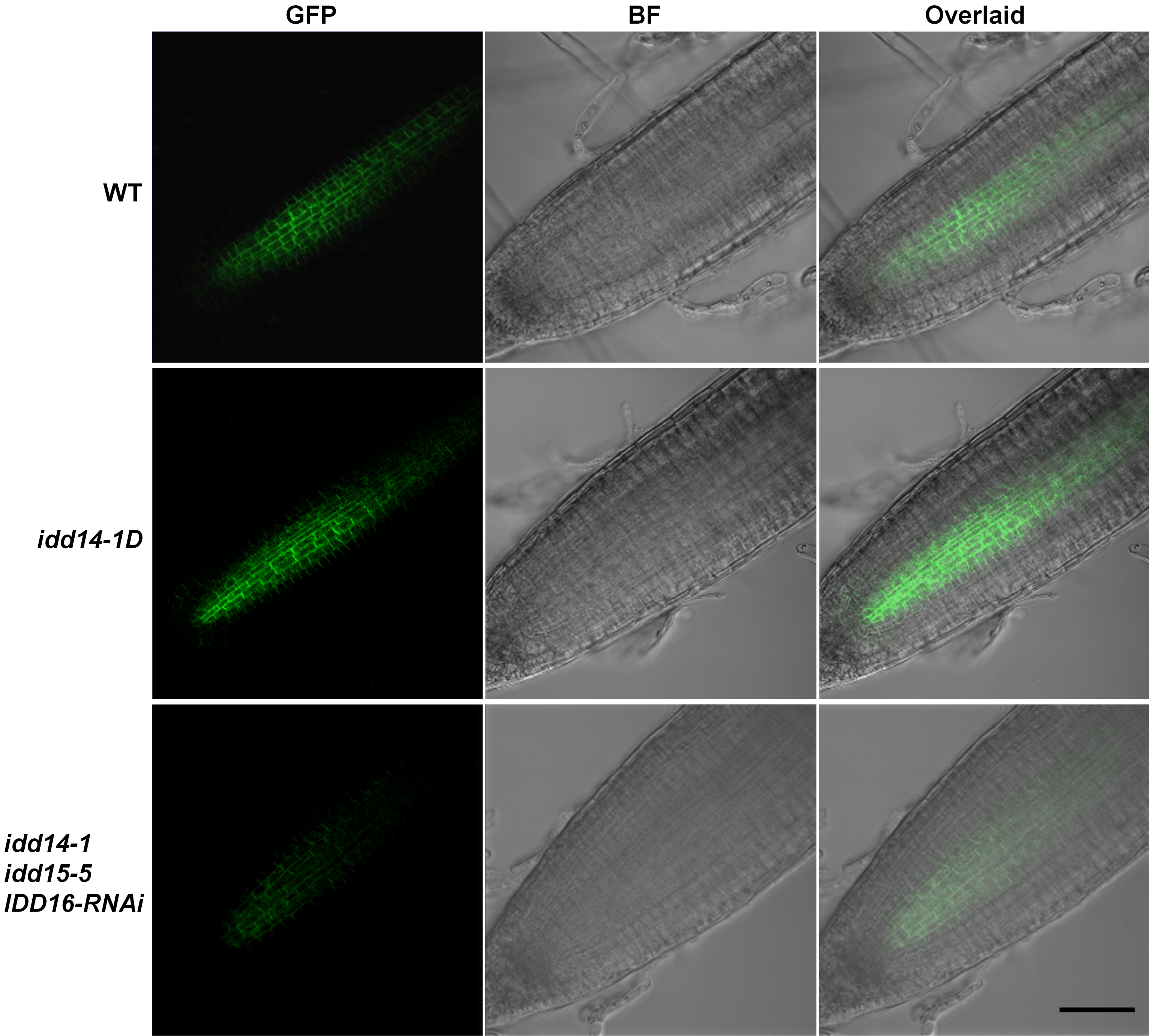

Supplement: Figure S6 — PIN1 accumulation in gain- and loss-of-function idd mutants. GFP fluorescent signals in primary roots of WT, idd14-1D, and idd triple mutant plants containing a pPIN1::PIN1-GFP construct. GFP fluorescence, bright fields (BF), and overlaid images are shown from left to right. The scale bar represents 50 µm. (TIF) [file pgen.1003759.s006.tif]

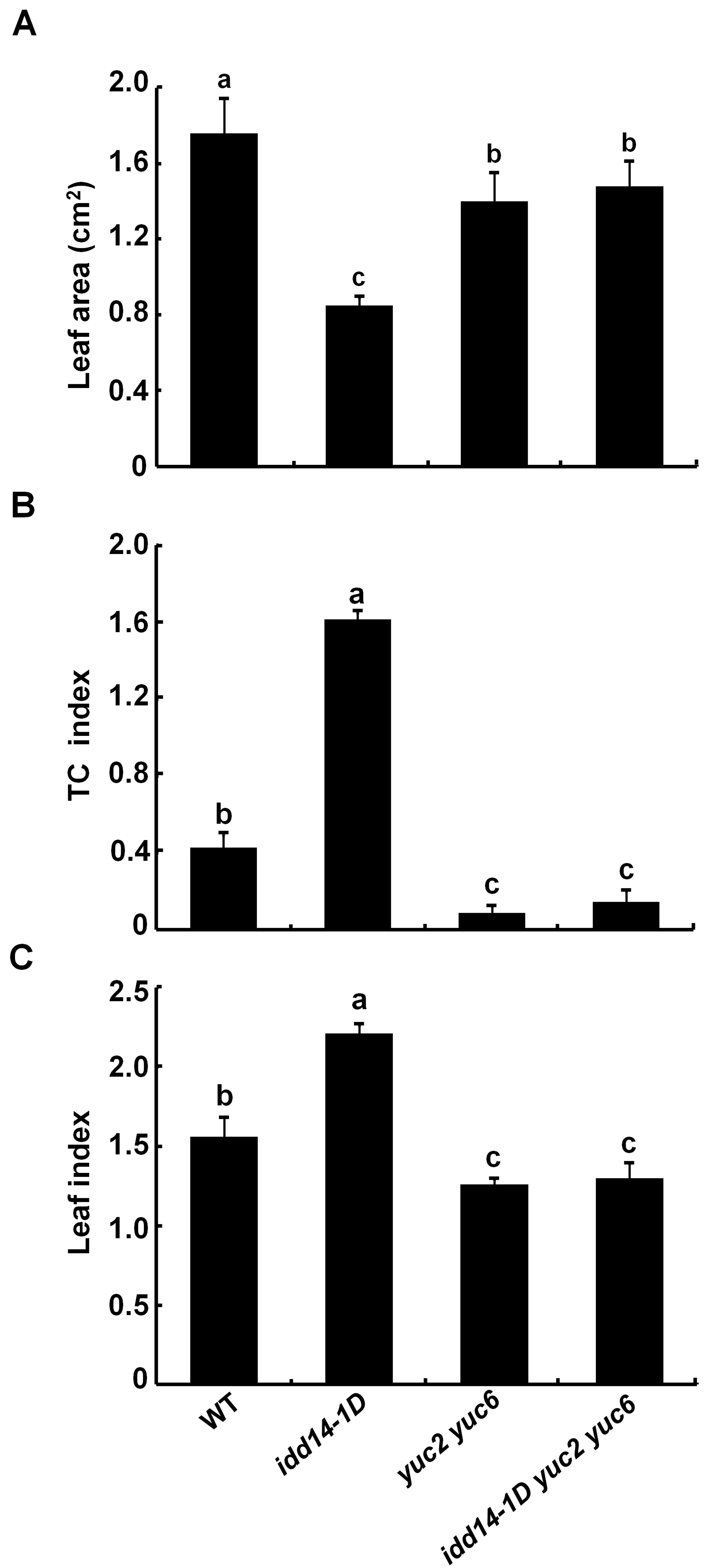

Supplement: Figure S7 — Suppression of idd14-1D phenotypes by yuc2 yuc6. (A–C) The blade areas (A), transverse curvature (TC) index (B), and leaf index (C) of WT, idd14-1D, yuc2 yuc6, and idd14-1D yuc2 yuc6 leaves. At least 10 sixth leaves from each genotype were used for determination of the leaf area, TC index, and leaf index, respectively. Data are shown as mean values ± one SD (one-way ANOVA test, P<0.05). (TIF) [file pgen.1003759.s007.tif]

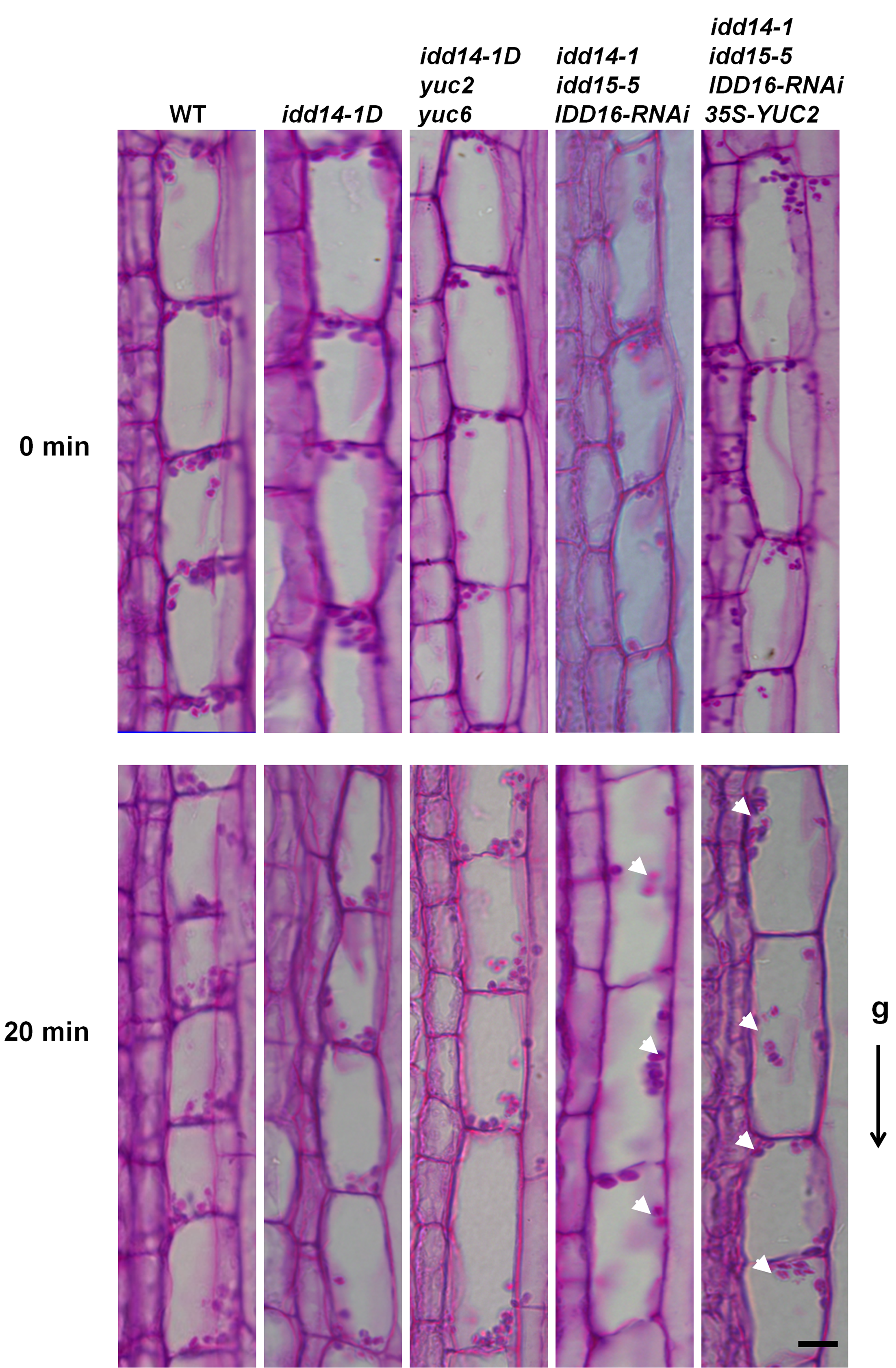

Supplement: Figure S8 — Alteration of auxin biosynthesis does not affect amyloplast sedimentation in idd mutants. Plants were gravistimulated by turning upside down for 0 or 20 min, and longitudinal sections of inflorescence stems were prepared and then stained with a periodic acid-Schiff kit. Arrowheads indicate the retarded movement of amyloplasts in the endodermal cells of the triple idd mutant and triple idd mutant carrying a p35S::YUC2 construct. The scale bar represents 10 µm. (TIF) [file pgen.1003759.s008.tif]
