# Supplementary material for: The Arabidopsis IDD14, IDD15, and IDD16 Cooperatively Regulate Lateral Organ Morphogenesis and Gravitropism by Promoting Auxin Biosynthesis and Transport
Source: PLoS Genet. 2013 Sep 5;9(9):e1003759. doi: 10.1371/journal.pgen.1003759 (PMC3764202; doi:10.1371/journal.pgen.1003759)
Supplement: Table S1 — Phenotypic characterization of cuf1-D plants. (DOC) [file pgen.1003759.s009.doc]

**Table S1. Phenotypic Characterization of *cuf1-D* Plants.**

| Measurements | Wild type | *cuf1-D* |
| --- | --- | --- |
| Days of bolting (day) | 24.9 ± 1.0 | 29.2 ± 1.1*** |
| Plant height (cm)^a^ | 23.4 ± 1.5 | 19.9 ± 1.0*** |
| Silique length (mm)^b^ | 12.8 ± 0.4 | 13.6 ± 0.6* |
| Pedicel length (mm) ^b^ | 5.3 ± 0.3 | 5.5 ± 0.2 |
| Primary root length (mm)^c^ | 21.2 ± 1.8 | 20.3 ± 1.8 |

^a^ 6-week-old plants were used for measurement of plant height.

^b^ 50-day-old plants were used to determine silique and pedicel length.

^c^ 7-day-old seedlings were used for examining the length of primary roots.

At least 10 plants for WT and homozygous *cuf1-D* were measured, and the data are shown as mean values ± one SD. The student’s *t*-test was performed (*P<0.05 and ***P<0.001)
