# Supplementary material for: The Arabidopsis IDD14, IDD15, and IDD16 Cooperatively Regulate Lateral Organ Morphogenesis and Gravitropism by Promoting Auxin Biosynthesis and Transport
Source: PLoS Genet. 2013 Sep 5;9(9):e1003759. doi: 10.1371/journal.pgen.1003759 (PMC3764202; doi:10.1371/journal.pgen.1003759)
Supplement: Table S2 — Primers used in this study. (DOC) [file pgen.1003759.s010.doc]

**Table S2. Primers Used in This Study.**

| Primer | Usage | Sequence |
| --- | --- | --- |
| pSK-LB2 | Genotyping | 5’-CCTCCATATTGACCATCATACTC |
| CUF1-P1 | Genotyping | 5’-TCATCACTATGCTCTTCCCTTAAT |
| CUF1-P2 | Genotyping | 5’-GCCTATAGGAACCAAAGTAAAATAC |
| GABI-LB1 | Genotyping | 5’-ATATTGACCATCATACTCATTGC |
| idd14-LP | Genotyping | 5’-ATCCCAACCCTCCAACCAC |
| idd14-RP | Genotyping | 5’-GATGCTCTATCACTCGCCTTTT |
| SALK-LBb1 | Genotyping | 5’-ATTTTGCCGATTTCGGAAC |
| idd15-LP | Genotyping | 5’-CAATGATGGCATAGTGCAATG |
| idd15-RP | Genotyping | 5’-GGAGAAGACACGACCACAGTC |
| IDD14cds-F | RT-PCR/Cloning | 5’-ATGATAGACTACGAGAGAAGCAATAC |
| IDD14cds-R | RT-PCR/ Cloning | 5’-CTATGAAGATGCTCTATCACTCGC |
| IDD15cds-F | RT-PCR/ Cloning | 5’-ATGAGAACAGATCAAGTGATGTTGTC |
| IDD15cds-R | RT-PCR/ Cloning | 5’-TTAAAAACCATTTTCCAACTCTCC |
| IDD16cds-F | RT-PCR/ Cloning | 5’-ATGATACATTACGAACAAAACAAC |
| IDD16cds-R | RT-PCR/ Cloning | 5’-TCACTCGCATTCTCCTTCAGTTGTC |
| IDD14gus-F | Cloning | 5’-CGGTCGACACTTCAATTTTCTTACAGTTCC |
| IDD14gus-R | Cloning | 5’-ACCCGGGCTCTCGTAGTCTATCATCG |
| IDD15gus-F | Cloning | 5’-GGTCGACTCTTCCCCTAACTATGTATGG |
| IDD15gus-R | Cloning | 5’-CCCGGGACTTGATCTGTTCTCATCAAT |
| IDD16gus-F | Cloning | 5’-CGGTCGACAACATGAGTAATTTCGAT |
| IDD16gus-R | Cloning | 5’-TCCCGGGTCGTAATGTATCATTGGAT |
| IDD15RNAiF-F | Cloning | 5’-TATCTAGACACCATCCGGCAACCTC |
| IDD15RNAiF-R | Cloning | 5’-TATCTGCAGGTGCTGATGTTCTTGCC |
| IDD15RNAiR-F | Cloning | 5’-TACTCGAGATCTCACCATCCGGCAACCT |
| IDD15RNAiR-R | Cloning | 5’-TAATCGATGTGCTGATGTTCTTGCCATTCC |
| IDD14GFP-F | Cloning | 5’-GACTCGAGATGATAGACTACGAGAGAAGC |
| IDD14GFP-R | Cloning | 5’-TAGGTACCTGAAGATGCTCTATCACTCGC |
| IDD16GFP-F | Cloning | 5’-ATGATACATTACGAACAAAACAACA |
| IDD16GFP-R | Cloning | 5’-CTCGCATTCTCCTTCAGTTGTC |
| YUC2cdna-F | Cloning | 5’-AATTCTTGAGAGATTTTCTTATAACAAA |
| YUC2cdna-R | Cloning | 5’-CAAATAAATATATACAAATGTGGACCAT |
| IDD14situ-F | In situ | 5’-CTTTGTTTCTCTCTCTTAACTCATCTTA |
| IDD14situ-R | In situ | 5’-TCCATTACCGTCGGGAAGA |
| IDD15situ-F | In situ | 5’-TGAGAGAGAAATAGGTTTTTGAAGAA |
| IDD15situ-R | In situ | 5’-TTCTTTTTGACTTTATTGTATTTGGAT |
| IDD16situ-F | In situ | 5’-CCTCCAATACCACCATCGTTA |
| IDD16situ-R | In situ | 5’-GACAAAGATACCACCTCTGCATC |
| ACT2-F | RT-PCR | 5’-GCCATCCAAGCTGTTCTCTC |
| ACT2-R | RT-PCR | 5’-GCTCGTAGTCAACAGCAACAA |
| GAPC-F | RT-PCR | 5’-TGGTCGTTTGGTTGCTAGAGT |
| GAPC-R | RT-PCR | 5’- AAGGTCGGACTTGTATTCGTG |
| IAA5-F | RT-PCR | 5’-GACTCGAAATCACCGAACTA |
| IAA5-R | RT-PCR | 5’-ATCTCCAGCAAGCATCCAA |
| IAA29-F | RT-PCR | 5’-TGCCTTAGAAATGGAGTTGG |
| IAA29-R | RT-PCR | 5’-CACAGTAGCCGTTGTTGGAT |
| qACT2-F | qRT-PCR | 5’- GCTCCTCTTAACCCAAAGGC |
| qACT2-R | qRT-PCR | 5’- CACACCATCACCAGAATCCAGC |
| qYUC1-F | qRT-PCR | 5’-CAAAGAAAGGAGCAAAGTTTATGG |
| qYUC1-R | qRT-PCR | 5’-CTGAAGCCAAGTAGGCACGTT |
| qYUC2-F | qRT-PCR | 5’-ACCCTACTTACCCTACAAAGCAACA |
| qYUC2-R | qRT-PCR | 5’-CTCACCCTCCATAACCCACAC |
| qYUC3-F | qRT-PCR | 5’-GATTTTGGCGGCGATGTT |
| qYUC3-R | qRT-PCR | 5’-GGGTTTGCTCCGTGATTGTAG |
| qYUC4-F | qRT-PCR | 5'-AAAACTCCCGTTCTTGATGTCG |
| qYUC4-R | qRT-PCR | 5'-TCTTTCCCATTCAGAAACTTTGC |
| qYUC5-F | qRT-PCR | 5’-GGGTTAACGGTCCTGTAATCGT |
| qYUC5-R | qRT-PCR | 5’-TCTGCTCTCTCCAATACCACAAAG |
| qYUC6-F | qRT-PCR | 5’-ATTTCCAATACAAGAGTTCCCTGAG |
| qYUC6-R | qRT-PCR | 5’-CATTGATGCTCCACTAATCCCAC |
| qYUC7-F | qRT-PCR | 5’-TGATTCTTGCCACTGGTTACAGA |
| qYUC7-R | qRT-PCR | 5’-CCTTCCCTTTCCACCCTTTT |
| qYUC8-F | qRT-PCR | 5’-TGTATGCGGTTGGGTTTACG |
| qYUC8-R | qRT-PCR | 5’-CAGAGCCTATGTCTTGTGCGAT |
| qYUC9-F | qRT-PCR | 5’-GGTTGGCTGGAGCATCAGTAGA |
| qYUC9-R | qRT-PCR | 5’-TTCGGTGACCCACATTTCTTCT |
| qYUC10-F | qRT-PCR | 5’-TCCTTTCGCCACCAAACTCT |
| qYUC10-R | qRT-PCR | 5’-AATCCCACCATTGATAACCTGAA |
| qYUC11-F | qRT-PCR | 5’-CGTCGATTAAGCGTATAGAAGGGA |
| qYUC11-R | qRT-PCR | 5’-CCAGTTGCGAAGACAATGGAGT |
| qTAA1-F | qRT-PCR | 5’-CCCCACTACACTCCCATCACTC |
| qTAA1-R | qRT-PCR | 5’-TCACCAATGCCCACCCAATAC |
| qTAR1-F | qRT-PCR | 5’-GCAACTTCTTTGGCAAAACCC |
| qTAR1-R | qRT-PCR | 5’-ATCGGTCTCCTCCTCTCGTCA |
| qTAR2-F | qRT-PCR | 5’-TTGGGGTTTCAAAGGACTCAC |
| qTAR2-R | qRT-PCR | 5’-TTTGCTGCTTGTTTCAATAGTTTC |
| qAUX1-F | qRT-PCR | 5’-GGGAGAAAGTGATTGGGATGC |
| qAUX1-R | qRT-PCR | 5’-CGACAGCGGAATTGATTGGA |
| qLAX3-F | qRT-PCR | 5’-ATGCTTACCTTTGCTCCTGCTC |
| qLAX3-R | qRT-PCR | 5’-ACCCGAACCCAACTACGAATAC |
| qPIN1-F | qRT-PCR | 5’-AAACGACGCAGGCTAAGGTG |
| qPIN1-R | qRT-PCR | 5’-CACTTGAAGGAAATGAGGGACC |
| qPIN2-F | qRT-PCR | 5’-GCTGGTCTTGGAATGGCTATGT |
| qPIN2-R | qRT-PCR | 5’-CATCGCAAACCCTGCTACTGA |
| qPIN3-F | qRT-PCR | 5’-GCGAGTGTGATGACAAGGCTG |
| qPIN3-R | qRT-PCR | 5’-AGAGCCCAAATAAGTCCAATGAG |
| qPIN4-F | qRT-PCR | 5’-ATTGCTTGTGGGAACTCTGTCG |
| qPIN4-R | qRT-PCR | 5’-AGGTCGCCGTGTAAGCCAAT |
| qPIN7-F | qRT-PCR | 5’-GGAGGAAACTCATAAGAAACCCA |
| qPIN7-R | qRT-PCR | 5’-ACATCCCACCTGAAAGCAACA |
| qABCB1-F | qRT-PCR | 5’-TTTCCGTGACCTTAGCCTTCG |
| qABCB1-R | qRT-PCR | 5’-CCTTATGTCTTTCCCGTCGATC |
| qABCB4-F | qRT-PCR | 5’-GAAATGTGAAGGTCCAATGAAGAA |
| qABCB4-R | qRT-PCR | 5’-GTAGAAACTGGCAGCGTAAGAAGA |
| qABCB19-F | qRT-PCR | 5’-ATTCCCACTTCTAGTCCTCGCTAA |
| qABCB19-R | qRT-PCR | 5’-GCTGCTACGGTTCTAATGTTACTGA |
| qPID-F | qRT-PCR | 5’-GTCTAAACTTTGCGCTCATTCGT |
| qPID-R | qRT-PCR | 5’-GCCGCTGGTTTGTTACTACTTCTA |
| qWAG1-F | qRT-PCR | 5’-ACGGAAGCGGTGTAGATTGGT |
| qWAG1-R | qRT-PCR | 5’-GCGCAGGGTTTGTTCTTTTGTA |
| qWAG2-F | qRT-PCR | 5’-TTCAGAGTTTGCGGCAGAGC |
| qWAG2-R | qRT-PCR | 5’-TATTCCGAACGCCCACCAG |
| qRCN1-F | qRT-PCR | 5’-AAGGGACAATGAGGCTGAAGTG |
| qRCN1-R | qRT-PCR | 5’-GAAGGATGTGCTGAATCGCAA |
| YUC2a-F | ChIP | 5’-TCCATTAAAATGTAAGTTCATAGGTCA |
| YUC2a-R | ChIP | 5’-TTTGTTTCTCCTTTTATAGCCTTACTA |
| YUC2b-F | ChIP | 5’-AGCTAATTCTTGGATCATACCGAGA |
| YUC2b-R | ChIP | 5’-AAACAATGAACCCACTAAAGAATCC |
| YUC2c-F | ChIP | 5’-TGCTTCTCTCTCCCCAAGTATTT |
| YUC2c-R | ChIP | 5’-AACCAGGGTAAATCTAATGATGAAAT |
| YUC3a-F | ChIP | 5’-CCTCAAATTCCACAGCCGTCTA |
| YUC3a-R | ChIP | 5’-TTTTATTCACATTTTGCGACGATTA |
| YUC3b-F | ChIP | 5’-GTCTGAACCGACCATAATCTCTCT |
| YUC3b-R | ChIP | 5’-ATCCTCGTCCAACAAATACAATAAT |
| YUC5a-F | ChIP | 5’-TGTACATGCACTAACTTCACAAATTAG |
| YUC5a-R | ChIP | 5’-TTAGGTCCATTTATTGTAAGTTTGTTT |
| YUC5b-F | ChIP | 5’-GTTAAGCAGTCAGCAGTCGGC |
| YUC5b-R | ChIP | 5’-TCTCCTTTTGAAACCGAATGAAC |
| YUC5c-F | ChIP | 5’-GCCTAAAATTGGCTTTTGATCTAGT |
| YUC5c-R | ChIP | 5’-TGGATTGGGAAAACTACTTGTCAT |
| YUC5d-F | ChIP | 5’-ACAATCTCAATATATACCATAAAGCAAT |
| YUC5d-R | ChIP | 5’-GCAAAAGTTCTTCAATAAGTGTGTG |
| YUC5e-F | ChIP | 5’-GAAGTGAAGATTCCTCCGACAGA |
| YUC5e-R | ChIP | 5’-GGGAAGGGCATTTTGGGTA |
| TAA1a-F | ChIP | 5’-TCTATTTAGAGATTTACGAAAGCGAC |
| TAA1a-R | ChIP | 5’-CAAAACTAAAAAGAAAAATTACAACAGA |
| TAA1b-F | ChIP | 5’-TTTTGTTTATTATTGAGTAATTCTTTTCA |
| TAA1b-R | ChIP | 5’-GATTAGAGAATAGTGTAGGTCGAAGTG |
| TAA1c-F | ChIP | 5’-CAATCTGGATCAGTACGTTTCTTCT |
| TAA1c-R | ChIP | 5’-CACCCATCTTCCTCCAGTATTCT |
| TAA1d-F | ChIP | 5’-GCCGTCCACGCACTCTCTT |
| TAA1d-R | ChIP | 5’-CAAAAAACAATCCCCACAAAAGT |
| PIN1a-F | ChIP | 5’-AAAAAAAGAGAGAGGCAAAAACAG |
| PIN1a-R | ChIP | 5’-ACATTCCAACATAATAGTGTGTGTGA |
| PIN1b-F | ChIP | 5’-AATCAGCAAACAGTTTTAGCTTCATTA |
| PIN1b-R | ChIP | 5’-GCCGTAATCATCTTTTGTTCGC |
| PIN1c-F | ChIP | 5’-ACGTTATGACGGCTATGGTTCC |
| PIN1c-R | ChIP | 5’-GGAGAGAATCTGCGGCGAG |
| PIN1d-F | ChIP | 5’-GTTTCCAGACACAGCAGGATCTAT |
| PIN1d-R | ChIP | 5’-AGGTCTAGGTGTCGCAGATAAGC |
| QQS-F | ChIP | 5’-TTTGTGATCCCTGACGCAGT |
| QQS-R | ChIP | 5’-TTGCTGCTGGAACCAGAGTT |
| ACT2Ch-F | ChIP | 5’-TAGTTAACACGAGGGAAAAGGCT |
| ACT2Ch-R | ChIP | 5’-AAAGTGAGGAGGACAACGAGACA |
|  |  |  |
